# DNA Methylation Impacts Gene Expression and Ensures Hypoxic Survival of *Mycobacterium tuberculosis*

**DOI:** 10.1371/journal.ppat.1003419

**Published:** 2013-07-04

**Authors:** Scarlet S. Shell, Erin G. Prestwich, Seung-Hun Baek, Rupal R. Shah, Christopher M. Sassetti, Peter C. Dedon, Sarah M. Fortune

**Affiliations:** 1 Department of Immunology and Infectious Diseases, Harvard School of Public Health, Boston, Massachusetts, United States of America; 2 Department of Biological Engineering and Center for Environmental Health Studies, Massachusetts Institute of Technology, Cambridge, Massachusetts, United States of America; 3 Department of Microbiology & Physiological Systems, University of Massachusetts Medical School, Worcester, Massachusetts, United States of America; Johns Hopkins School of Medicine, United States of America

## Abstract

DNA methylation regulates gene expression in many organisms. In eukaryotes, DNA methylation is associated with gene repression, while it exerts both activating and repressive effects in the Proteobacteria through largely locus-specific mechanisms. Here, we identify a critical DNA methyltransferase in *M. tuberculosis*, which we term MamA. MamA creates N^6^-methyladenine in a six base pair recognition sequence present in approximately 2,000 copies on each strand of the genome. Loss of MamA reduces the expression of a number of genes. Each has a MamA site located at a conserved position relative to the sigma factor −10 binding site and transcriptional start site, suggesting that MamA modulates their expression through a shared, not locus-specific, mechanism. While strains lacking MamA grow normally *in vitro*, they are attenuated in hypoxic conditions, suggesting that methylation promotes survival in discrete host microenvironments. Interestingly, we demonstrate strikingly different patterns of DNA methyltransferase activity in different lineages of *M. tuberculosis*, which have been associated with preferences for distinct host environments and different disease courses in humans. Thus, MamA is the major functional adenine methyltransferase in *M. tuberculosis* strains of the Euro-American lineage while strains of the Beijing lineage harbor a point mutation that largely inactivates MamA but possess a second functional DNA methyltransferase. Our results indicate that MamA influences gene expression in *M. tuberculosis* and plays an important but strain-specific role in fitness during hypoxia.

## Author Summary

Tuberculosis is a disease with a devastating impact on public health, killing over 1.5 million people each year around the globe. Tuberculosis is caused by the bacterium *Mycobacterium tuberculosis*, which over millennia has evolved the ability to survive and persist for decades in the harsh environment inside its human host. Regulation of gene expression is critical for adaptation to stressful conditions. To successfully tackle *M. tuberculosis*, we therefore need to understand how it regulates its genes and responds to environmental stressors. In this work, we report the first investigation of the role of DNA methylation in gene regulation and stress response in *M. tuberculosis*. We have found that DNA methylation is important for survival of hypoxia, a stress condition present in human infections, and furthermore that DNA methylation affects the expression of several genes. In contrast to methylation-regulation systems reported in other bacteria, in which the effects of methylation vary from one gene to the next, *M. tuberculosis* appears to use a concerted mechanism to influence multiple genes. Our findings identify a novel mechanism by which *M. tuberculosis* modulates gene expression in response to stress.

## Introduction

*Mycobacterium tuberculosis* is a pathogen of tremendous global significance, causing 9 million cases of tuberculosis annually and latently infecting up to a third of the world's population [1]. Untreated, *M. tuberculosis* can persist for decades in the infected host. Over such timescales, the bacterium must tune gene expression patterns to match conditions in the host environment, including hypoxia, nutrient deprivation, and low pH, and maintain these adaptations over long periods of time.

How might *M. tuberculosis* durably maintain gene expression patterns? While eukaryotes use a variety of mechanisms to heritably ensure expression states, DNA methylation is the only known mechanism by which prokaryotes might achieve epigenetic inheritance. Both adenine and cytosine can be methylated in DNA, resulting in N^6^-methyladenine, N^4^-methylcytosine, and 5-methylcytosine (accurately termed N^6^-methyl-2′deoxyadenosine, N^4^-methyl-2′deoxycytidine, and 5-methyl-2′deoxycytidine, and abbreviated here as N^6^-MdA, N^4^-MdC, and 5-MdC, respectively). Cytosine methylation is an important mechanism of repressing gene expression in higher eukaryotes and recent reports suggest that 5-MdC has regulatory roles in prokaryotes [2], [3]. However, in prokaryotes N^6^-MdA is the best-characterized epigenetic regulator of gene expression [4]–[9].

Regulation of gene expression by adenine methylation has been described mainly in the Proteobacteria where it is primarily mediated by the Dam methyltransferase in the Gammaproteobacteria and CcrM in the Alphaproteobacteria, although other methyltransferases of unknown function have been identified [5], [10]. Dam-mediated methylation has pleiotropic roles that include directing DNA mismatch repair, suppressing transposition, and regulating genes involved in cell cycle timing and antigenic variation [5]–[9], [11]–[17]. In *Escherichia coli*, genetic disruption of *dam* causes a modest growth defect [18], an increased mutation rate [19], [20], and numerous gene expression changes [21]–[23]. Some of these expression changes result directly from the methylation state of a given promoter, but most seem to reflect the downstream consequences of cell cycle changes and perturbed DNA repair [7]–[9], [11], [15]–[17], [24]–[28]. Even where Dam methylation has been shown to regulate gene expression directly, the mechanistic details are highly locus-specific [7], [17], [29], [30]. There are several known transcriptional repressors that bind DNA in a methylation state dependent manner. Methylation may permit or prevent repressor binding, depending on the repressor and the spatial relationship between the Dam site and other promoter elements. However, the pleiotropic roles of Dam methylation in cell cycle regulation and DNA repair make it difficult to distinguish between direct and indirect effects on gene expression. Furthermore, over half of the ORFs in the *E. coli* genome have two or more Dam sites in the 500 base pair region upstream [21], making the presence of Dam sites a poor indicator of Dam-mediated regulation.

Virulent *M. tuberculosis* has been reported to contain both N^6^-MdA and 5-MdC [31]. However, there are no predicted *dam* or *dcm* homologues in the genome and canonical Dam and Dcm sites are not methylated [31], [32]. Van Soolingen and colleagues identified a site in the *lppC* gene that was protected from restriction digest in clinical *M. tuberculosis* strains [33] and predicted this to be due to DNA methylation. However, nothing further was known about the mechanism or functional consequences of DNA methylation in *M. tuberculosis*.

Interestingly, the extent of *lppC* protection differed among strains from the different phylogeographic lineages of *M. tuberculosis*, with strains of the Beijing lineage showing reduced *lppC* protection compared to strains from other lineages [33]. The various lineages of *M. tuberculosis* are associated with different epidemiological characteristics. Most notably, strains of the Beijing lineage appear to be increasing in prevalence globally, suggesting that this lineage has a competitive advantage in the modern world [34]–[36]. While the success of the Beijing lineage is likely multifactorial, some of its unique characteristics have been hypothesized to arise from differences in regulatory circuitry that may alter adaptation to specific host environments [35], [37]–[40].

Based on these findings, we hypothesized that DNA methylation might regulate gene expression in *M. tuberculosis*, with functional significance in specific host environments or genetic contexts. We identify a methyltransferase, MamA (M.MtuHIII according to systematic DNA methyltransferase nomenclature [41]), and show that it methylates a six base pair sequence in the *M. tuberculosis* genome in a strain specific manner. We demonstrate that MamA methylation affects expression of several genes. Using a novel approach to map the transcriptional start sites of these genes we demonstrate that in each case, a methylation site overlaps with the sigma factor binding site in an identical configuration. Importantly, we show that loss of MamA reduces the ability of *M. tuberculosis* to survive in hypoxia, a stressor thought to mimic the environment that the bacterium encounters in the human host.

## Results

### A putative methylation site exhibits strain-dependent variability in restriction digest susceptibility

**Figure 1:**
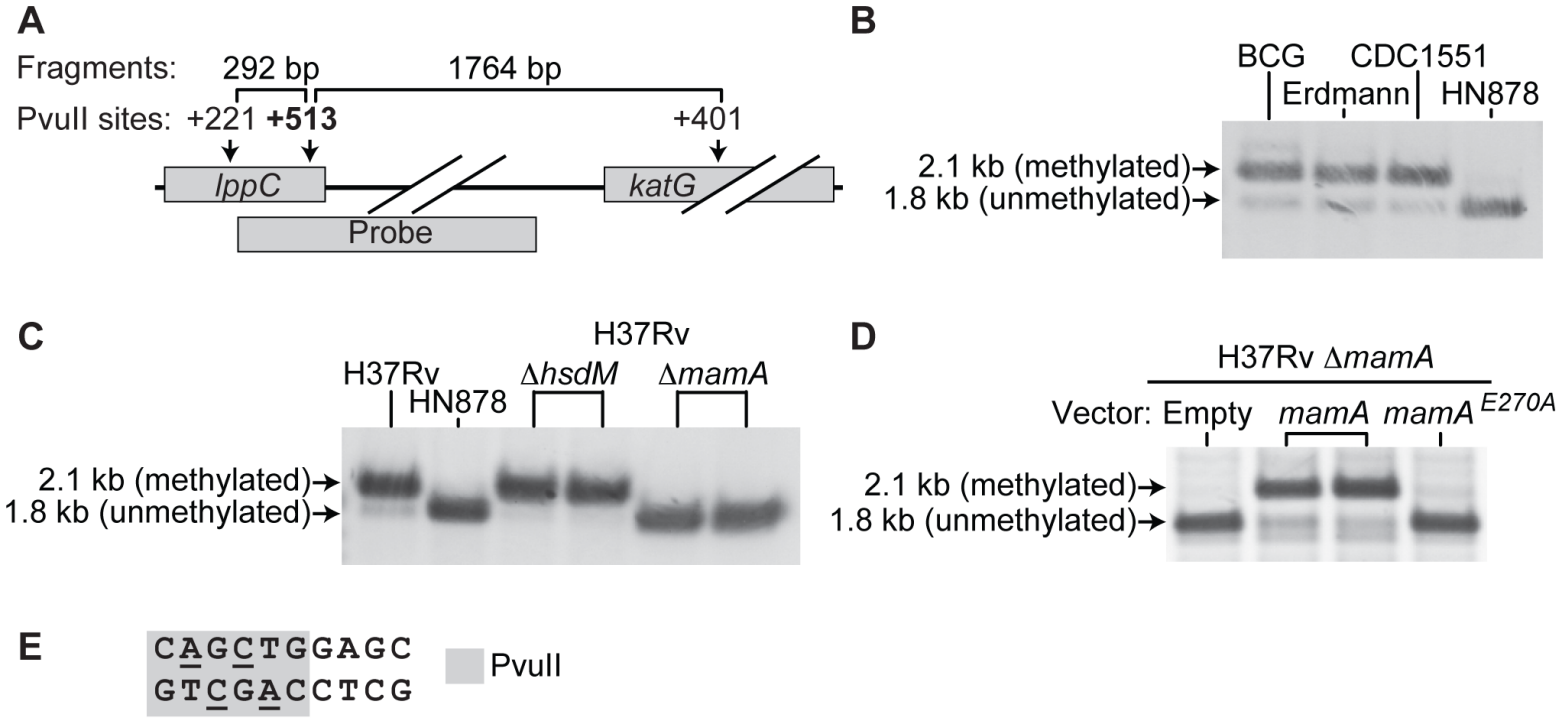
MamA is a DNA methyltransferase that protects *lppC* from endonucleolytic cleavage. (A) Southern blotting strategy to assess the status of a PvuII site near the 3′ end of *lppC*. Genomic DNA was digested with PvuII and analyzed by Southern blot with a probe hybridizing as shown. Fully cleaved DNA generates a 1.8 kb product, while protected DNA produces a 2.1 kb product. (B) DNA from the vaccine strain *M. bovis* BCG and from *M. tuberculosis* strains of the Euro-American lineage (Erdmann and CDC1551) is partially protected from PvuII cleavage while DNA from a Beijing lineage strain (HN878) is not. (C) Genetic deletion of *mamA* abrogates protection of *lppC*, while deletion of *hsdM* does not affect protection. (D) Protection is restored by complementation of a *ΔmamA* strain with an ectopic copy of *mamA*, but not by empty vector or *mamA^E270A^*. (E) Sequence context of the assayed PvuII site. Underlined bases are predicted to block PvuII if methylated.

In order to investigate the determinants of DNA methylation in the *M. tuberculosis* genome, we began by examining a site in the *lppC* gene that had been previously reported to be protected from restriction enzyme cleavage [33]. Consistent with the published data, we confirmed that this site was largely protected from cleavage by PvuII in *M. tuberculosis* strains from the Euro-American lineage and the vaccine strain *M. bovis* BCG (Figure 1, A and B), but was fully susceptible to PvuII in strain HN878, a member of the Beijing lineage of *M. tuberculosis* (Figure 1, B and C). As the PvuII recognition sequence was present in all strains, it had been postulated that differential methylation was the most likely explanation for the variable PvuII cleavage [33]. A 10 base pair sequence containing the PvuII recognition site was shown to be protected from PvuII cleavage [33]; methylation of the adenine residues within this sequence is expected to block PvuII cleavage [42] and the effects of cytosine methylation are unknown (Figure 1E).

### *Rv3263* encodes the active DNA methyltransferase MamA

There are two predicted DNA methyltransferases encoded in the *M. tuberculosis* genome, neither of which is associated with a cognate restriction endonuclease. To determine if either of these methyltransferases was responsible for the DNA modification at the *lppC* site, we constructed unmarked deletion mutants of these genes in H37Rv, a commonly used lab strain of *M. tuberculosis* that belongs to the EuroAmerican lineage. Deletion of *Rv3263* abolished protection of the *lppC* site from PvuII cleavage (Figure 1C). In contrast, deletion of *hsdM* did not affect protection of this site. Complementation of the *Rv3263* deletion strain with an ectopic copy of the gene restored protection (Figure 1D and Figure S1). The *Rv3263* gene product from H37Rv will be called M.MtuHIII according to standard DNA methyltransferase nomenclature [41]. As systematic methyltransferase names are strain-specific, we have also chosen a generic name that can be applied to all *M. tuberculosis* strains. We therefore refer to *Rv3263* and its gene product as *mamA* and MamA, respectively (Mycobacterial adenine methyltransferase). MamA is conserved in relatives of *M. tuberculosis* including *M. bovis* BCG (Figure 1), the pathogens *M. leprae* and *M. avium*, and the saprophyte *M. smegmatis* (TB Database, [43]).

### Sequence trace comparison reveals a six base pair recognition site for adenine methylation by MamA

**Figure 2:**
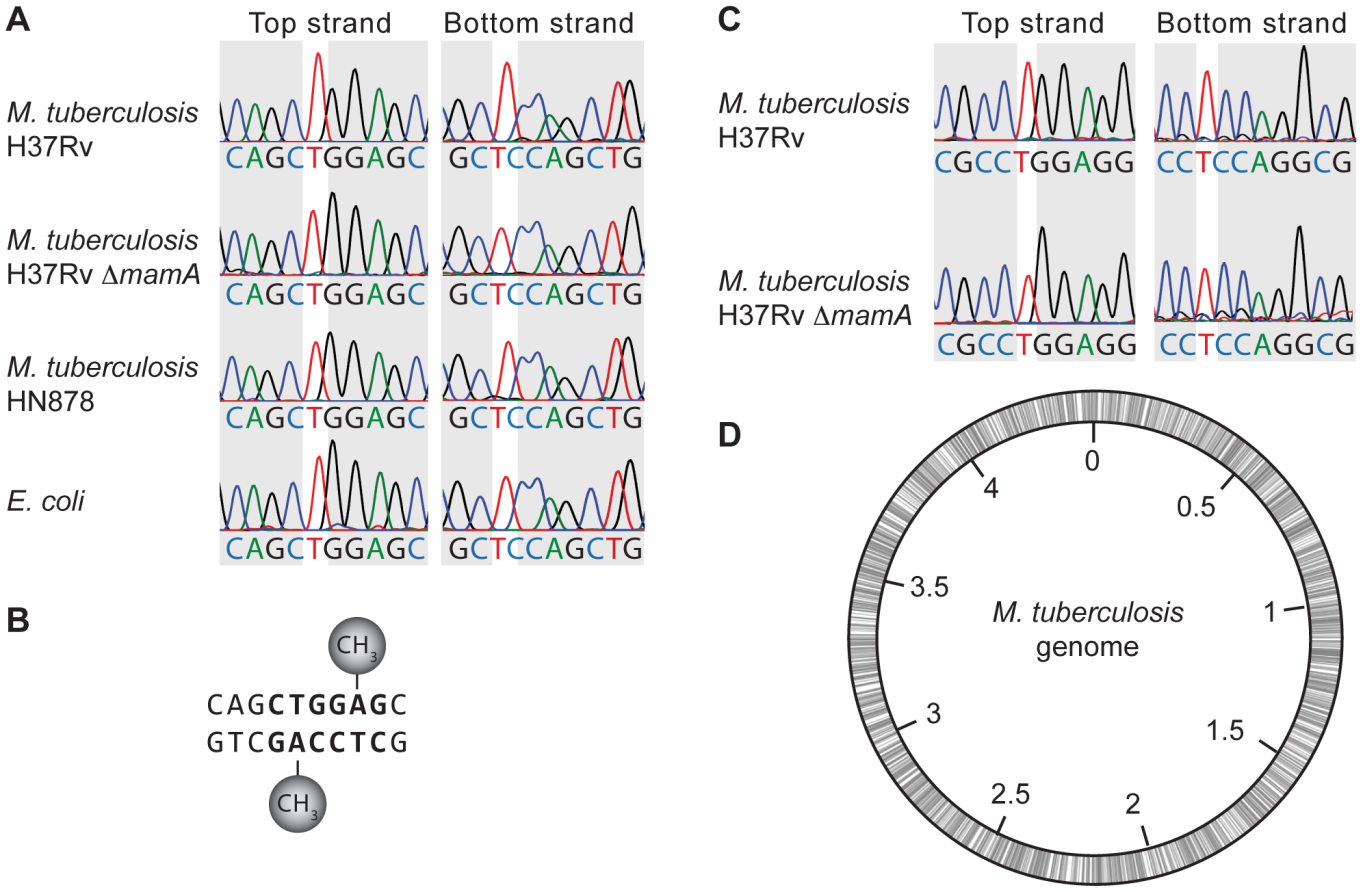
Sequence trace comparison identifies the target base and minimal recognition sequence of MamA. Plasmids containing putative MamA-recognition motifs were propagated in the indicated bacterial strains, isolated and sequenced. Sequence traces shown are representative of at least 2–3 biological replicates. (A) The 10 base pair sequence shown in Figure 1E supports methylation of one adenine on each strand in wildtype H37Rv, as evidenced by increased thymine peak areas relative to the identical sequence context in *E. coli* and methylation-deficient strains of *M. tuberculosis*. See Figure S2 for quantification of peak areas. (B) Schematic depiction of the positions of N^6^-methyladenine residues. (C) A six base pair core sequence is sufficient to direct MamA-mediated methylation (bold in panel B). See Table S1 for a complete list of tested sequences. (D) Positions of MamA recognition sequences are shown schematically on the 4.4 Mb *M. tuberculosis* genome.

To identify the base that MamA methylates, we constructed an episomal plasmid containing the 10 base pair sequence sufficient to enable protection from PvuII cleavage and propagated the plasmid in both wildtype *M. tuberculosis* and the *mamA* deletion mutant. We then assessed the methylation status of the 10 base pair sequence using sequence trace comparison. This method is based on differing incorporation of dye terminator nucleotides complementary to methylated adenine or cytosine residues in conventional Sanger sequencing, allowing methylation status to be inferred by comparing sequencing traces from identical sequences of DNA propagated in the presence and absence of the methyltransferase [44], [45]. The change in nucleotide incorporation depends on the methylated base in the template: N^6^-MdA results in increased incorporation of dideoxythymidine nucleotides yielding higher thymine peaks while 5-MdC and N^4^-MdC result in less and more dideoxyguanosine incorporation, respectively, and thus lower and higher guanine peaks [10], [44], [45]. We propagated the plasmid in methylation-proficient and methylation-deficient *M. tuberculosis* and *E. coli*, then purified and sequenced it. Representative sequence traces are shown in Figure 2A. The thymine peak in position 5 of the top strand sequence showed increased intensity in plasmid isolated from the methylation-proficient *M. tuberculosis* strain H37Rv, relative to the equivalent peak in sequences of plasmid isolated from *E. coli*, H37Rv *ΔMamA*, and *M. tuberculosis* strain HN878. Similarly, the thymine peak in position 3 of the opposite strand was relatively higher in plasmid from H37Rv. Quantification of differences in peak area is shown in Figure S2. These alterations in relative peak height reflect increases in dideoxythymidine incorporation, suggesting presence of N^6^-MdA in the complementary templates isolated from H37Rv (Figure 2B).

We also noted a reduction in the height of the guanine peak following the elevated thymine peak in H37Rv-derived DNA (Figure 2A, “top strand” and Figure S2A). This reflects decreased dideoxyguanosine incorporation and would be consistent with the presence of 5-MdC in the template, but bisulfite sequencing of H37Rv-derived plasmid indicated that no methylcytosine was present (data not shown). The peak height difference is therefore likely a result of the preceding N^6^-MdA causing an effective change in sequence context. Similar alterations in the incorporation of nucleotides neighboring the base complementary to the site of methylation have been observed previously [10], [46].

To determine the minimal recognition sequence required for methylation by MamA, we systematically mutated the 10 base pair sequence shown in Figure 2B and performed sequence trace comparison on the resulting plasmids. A central core of six base pairs (bold in Figure 2B) was sufficient to direct methylation in H37Rv (Figure 2C). Any further changes to this six base pair sequence abrogated methylation (Table S1). The MamA recognition site “CTGGAG” is predicted to be present in 1947 locations in the H37Rv genome. The sites are distributed across the genome, without any obvious skew with respect to the origin of replication (Figure 2D). Interestingly, there is a strong bias regarding the orientations of MamA sites relative to the coding strand within open reading frames. Of the 1816 times that MamA sites occur within annotated coding regions, the sequence reading “CTGGAG” is located on the coding strand in 1511 cases, while it is located on the non-coding strand in only 305 cases (p<0.0001, Chi square test with Yates correction). This may be at least partially a result of codon bias, as the codons “CTG” and “GAG” are both favored in *M. tuberculosis* while “CTC,” “TCC,” and “CCA” are all relatively disfavored [47]. Two other bacterial DNA methyltransferases, M.GsuI and M.BpmI, are known to recognize an identical sequence to MamA; however, the roles of these enzymes are not known so they did not provide clues regarding the function of MamA (Rebase, [42]).

### MamA is the predominant DNA methyltransferase in *M. tuberculosis*, H37Rv

**Figure 3:**
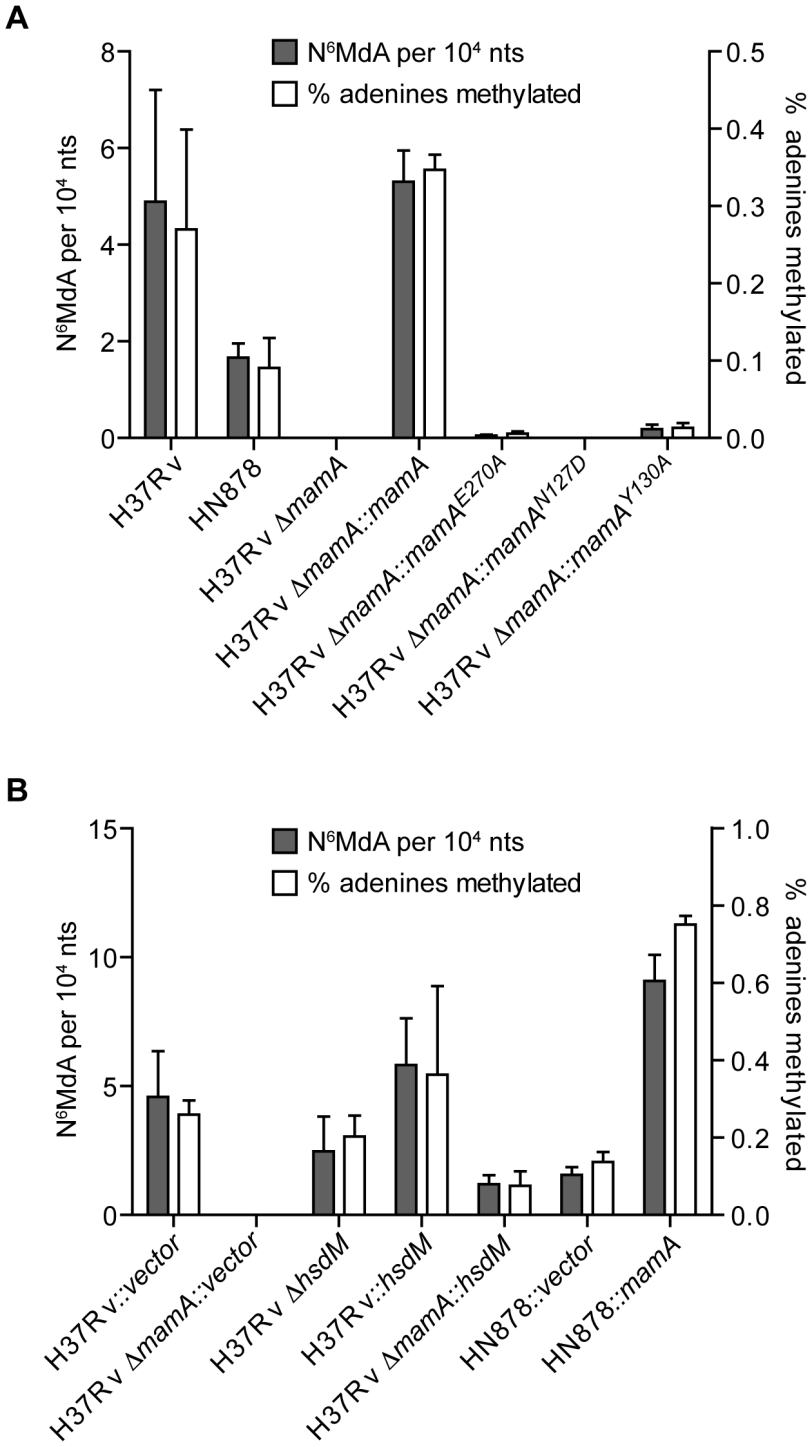
Quantitation of total N^6^-MdA content in *M. tuberculosis*. Genomic DNA from the indicated strains was digested to individual nucleosides and methylation content determined by liquid chromatography-coupled tandem mass spectrometry. Results are expressed as the amount of N^6^-MdA per nucleotide (left axis) and percentage of adenines that are methylated in each genome (right axis). Each represents at least three biological replicates. Outliers were removed using Grubbs criteria and error bars represent ± standard deviation. (A) Analysis of the contribution of wildtype and mutant forms of MamA to total adenine methylation levels in strain H37Rv. (B) Analysis of the contributions of MamA and HsdM to total adenine methylation levels in Euro-American (H37Rv) and Beijing (HN878) strain backgrounds.

To investigate the role of MamA within the broader DNA methylation landscape of *M. tuberculosis*, we defined the spectrum of methylated nucleobases in *M. tuberculosis* DNA using liquid chromatography-coupled tandem mass spectrometry (LC-MS/MS). Genomic DNA was enzymatically digested to individual nucleosides and subject to LC- MS/MS to quantify N^6^-MdA, 5-MdC, N^4^-MdC, and 5-hydroxymethyl-2′deoxycytidine (5-HMdC). In DNA from wildtype H37Rv, N^6^-MdA occurred at a rate of 4.9+/−2.2 per 10^4^ nucleotides (nts) (0.27% of adenosines) (Figure 3A), which would yield 2048+/−910 N^6^-MdA per strand in the 4.4 million bp genome, correlating well with the expected 1947 MamA sites (+/− denotes SD). Deletion of *mamA* reduced N^6^-MdA to less than five N^6^-MdA per genome strand. Taken together, these data indicate that MamA is the major adenine methytransferase active in H37Rv. Complementation of H37Rv *ΔmamA* with *mamA* restored N^6^-MdA to wildtype levels. 5-MdC, N^4^-MdC, and 5-HMdC were not detected in any of the *M. tuberculosis* strains (limits of detection were approximately five per 10^8^ nts for 5-MdC and N^4^-MdC, and one per 10^5^ nts for 5-HMdC). The absence of 5-MdC was somewhat surprising given that this modification has been previously reported in H37Rv [31], [32]. However, it is consistent with the absence of a predicted cytosine methyltransferase in the genome.

To confirm that MamA is itself a DNA methyltransferase (rather than an activator of other methyltransferases, for example), we made point mutations predicted to disrupt the MamA active site. MamA is predicted to be a Type II DNA methyltransferase with architecture of the gamma subtype [48], and is therefore homologous to the well-characterized methyltransferases M.EcoKI and M.TaqI. Biochemical studies of these and other adenine methyltransferases demonstrate that mutations of key residues in the S-adenosylmethionine binding site inhibit methyltransferase activity without disrupting overall protein structure [49]–[54]. We therefore mutated these key residues and expressed constructs encoding MamA^N127D^ and MamA^Y130A^ in H37Rv *ΔmamA*. We then assessed the effects of these mutations on DNA methylation using LC-MS/MS analysis. Both mutants displayed little or no adenine methylation (Figure 3A), indicating that the predicted active site of MamA is critical to its ability to confer methylation.

### MamA and HsdM are differentially active in Beijing lineage vs. Euro-American lineage strains

We sought to understand the loss of adenine methylation at the PvuII site in *lppC* in Beijing lineage strains of *M. tuberculosis*. Sequencing of *mamA* revealed that the Beijing lineage strain HN878, and all modern Beijing lineage strains for which genome sequences are publicly available, have a point mutation of nucleotide A809C causing the amino acid substitution Glu270Ala. While Glu270 is not located in the predicted active site, it is part of a conserved motif, suggesting that a non-conservative mutation to Ala could disrupt enzyme activity. Consistent with this hypothesis, *mamA^E270A^* failed to restore protection from PvuII cleavage in an H37Rv *ΔmamA* strain (Figure 1D). LC-MS/MS analysis revealed that H37Rv harboring *mamA^E270A^* had N^6^-MdA levels that were 50–100 fold lower than the wildtype parent (Figure 3A).

Interestingly, strain HN878 had only a 3-fold reduction in N^6^-MdA compared to H37Rv, despite harboring the *mamA^E270A^* allele (Figure 3A). This suggested that HN878 has a substantial amount of MamA-independent adenine methylation, in contrast to H37Rv. We therefore further examined the contributions of the two predicted DNA methyltransferases, MamA and HsdM, to total adenine methylation levels in the two strain backgrounds. In H37Rv and most members of the Euro-American lineage of *M. tuberculosis*, *hsdM* contains a mutation resulting in the amino acid change Pro306Leu in the active site, which is predicted to abolish HsdM activity [48], [55]. Indeed, LC-MS/MS analysis of H37Rv *ΔhsdM* demonstrated that deletion of *hsdM* did not reduce levels of N^6^-MdA suggesting that in H37Rv, *hsdM* does not appreciably contribute to the N^6^-MdA content of the genome. Consistent with the idea that a Pro306Leu mutation is responsible for the lack of detectable HsdM activity in H37Rv, reintroduction of a wildtype Pro306 allele of *hsdM* to H37Rv *ΔmamA* significantly increased N^6^-MdA levels (Figure 3B). Since HN878 naturally encodes a wildtype Pro306 allele of *hsdM*, the excess N^6^-MdA in HN878 relative to H37Rv *mamA^E270A^* is likely to reflect greater HsdM activity in HN878 as compared to H37Rv.

We also predicted that complementing HN878 with a wildtype Glu270 allele of *mamA* would increase total N^6^-MdA levels. Interestingly, restoration of wildtype MamA to HN878 resulted in a quantitatively greater increase in N^6^-MdA than expected based on the effect of complementing H37Rv *ΔmamA* with the same construct expressing *mamA* (Figure 3). These data suggest that strain genetic background affects expression and/or activity of individual methyltransferases.

### Global expression profiling reveals differential gene expression in *ΔmamA* strains

**Figure 4:**
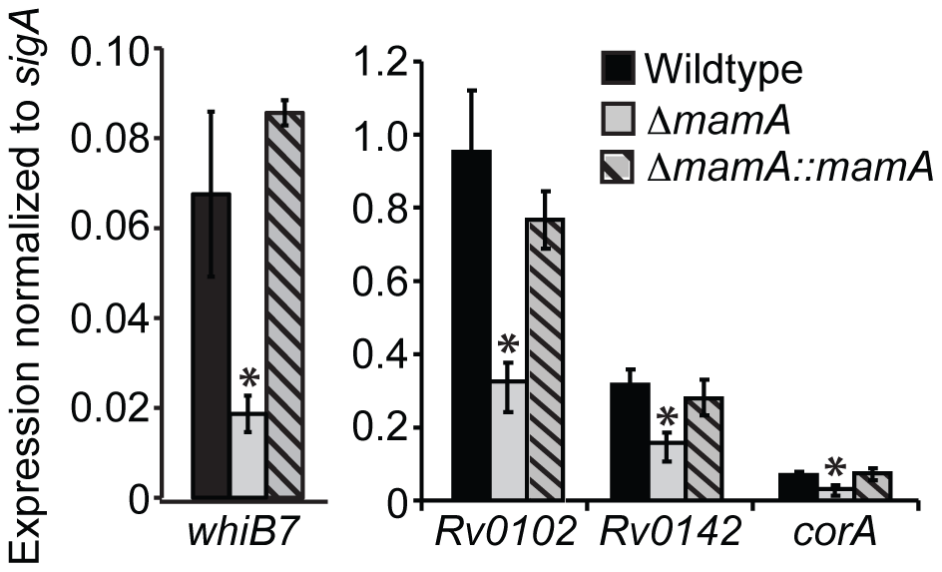
Several genes have lower expression levels in a *ΔmamA* strain. Expression of each gene was determined by quantitative PCR in the indicated H37Rv-derived strains and is displayed as a relative value compared to expression of the housekeeping gene *sigA* in the same strain. Values shown are the mean of three technical replicates. Error bars denote standard deviation. (*) denotes P<0.05 compared to the wildtype and complemented strains (ANOVA with Tukey's post test). The wildtype and complemented strains were not significantly different from each other for any of the genes tested. The experiment was performed using RNA from different cultures than those used to prepare RNA for microarrays.

As DNA methylation regulates gene expression in other organisms, we sought to determine if MamA serves a similar function in *M. tuberculosis*. We used an Affymetrix microarray platform to perform global transcriptional profiling of triplicate log-phase cultures of wildtype H37Rv, *ΔmamA*, and complemented strains (Table S2 for complete dataset; GEO accession number GSE46432). Table 1 lists genes with expression differences of 1.5-fold or greater between wildtype H37Rv and either of the other two strains. Because we saw only a modest number of expression differences of limited magnitude, we felt that the microarray experiment was best used as a hypothesis-generating tool. Recognizing that small changes in a bulk expression assay may reflect larger changes in heterogeneous subpopulations of bacteria, we hypothesize that such apparently subtle changes might be functionally important. Several genes showed lower expression in *ΔmamA* compared to wildtype and complemented strains and had MamA sites in the region upstream of their annotated start codons (Table 1). These genes were considered to be candidates whose expression might be directly regulated by DNA methylation. Other genes showed altered expression only in the complemented strain relative to the wildtype and *ΔmamA* strains. These genes were located in the vicinity of the integrating complementation vector and their expression changes were thus likely be a result the strain construction strategy and not related to methylation status. *Rv0102*, *Rv0142*, *corA*, *whiB7*, and the *Rv3083* operon were the strongest candidate methylation-affected genes. We therefore re-tested their expression levels by quantitative PCR (qPCR), using RNA derived from independent cultures, and confirmed that the *ΔmamA* strain had significantly reduced expression of *Rv0102*, *Rv0142*, *corA*, and *whiB7* (Figure 4).

**Table 1:** Genes with expression differences of 1.5-fold or greater in Δ*mamA* or complemented strains compared to the wildtype parent (H37Rv) in aerobic growth conditions.

		Log2 expression ratio		*p* a		
Gene	Symbol	Δ*mamA*/wildtype	Δ*mamA::mamA*/wildtype	Raw	FDR^b^	Distance (bp) to upstream MamA site^c^
Rv3263	*mamA*	−4.05	2.51	2.0×10^−12^	8.2×10^−9^	3213
Rv0142		−1.32	−0.16	4.0×10^−6^	4.2×10^−3^	143
Rv1239c	*corA*	−0.80	−0.22	0.037	1.0	43
Rv3197A	*whiB7*	−0.75	0.02	0.00067	0.31	336
Rv3083		−0.72	−0.19	0.0049	0.97	8
Rv0102		−0.72	0.03	0.0011	0.41	3
Rv3085		−0.68	−0.12	0.011	1.0	8^d^
Rv3084	*lipR*	−0.62	−0.06	0.026	1.0	8^d^
Rv3378c		−0.59	−0.07	0.038	1.0	1067
tRNA-pro	*proU*	−0.15	−1.91	0.00034	0.24	2480 or 6630^e^
Rv2463	*lipP*	0.016	−2.62	8.18×10^−9^	1.7×10^−5^	2544 or 476^e^
tRNA-gly^f^	*glyV*	−0.0089	−2.66	2.54×10^−7^	0.00035	2430

a ANOVA.

b Method of Benjamini and Hochberg.

c Distance from start of ORF to nearest upstream MamA site.

d Distance from start of first ORF in operon (Rv3083) to nearest upstream MamA site.

e Distances in wildtype/Δ*mamA* and complemented strains, respectively; complementation vector integrates in this region.

f Complementation vector integrates in this gene.

### Transcriptional start site (TSS)-mapping suggests direct modulation of gene expression by MamA methylation

**Figure 5:**
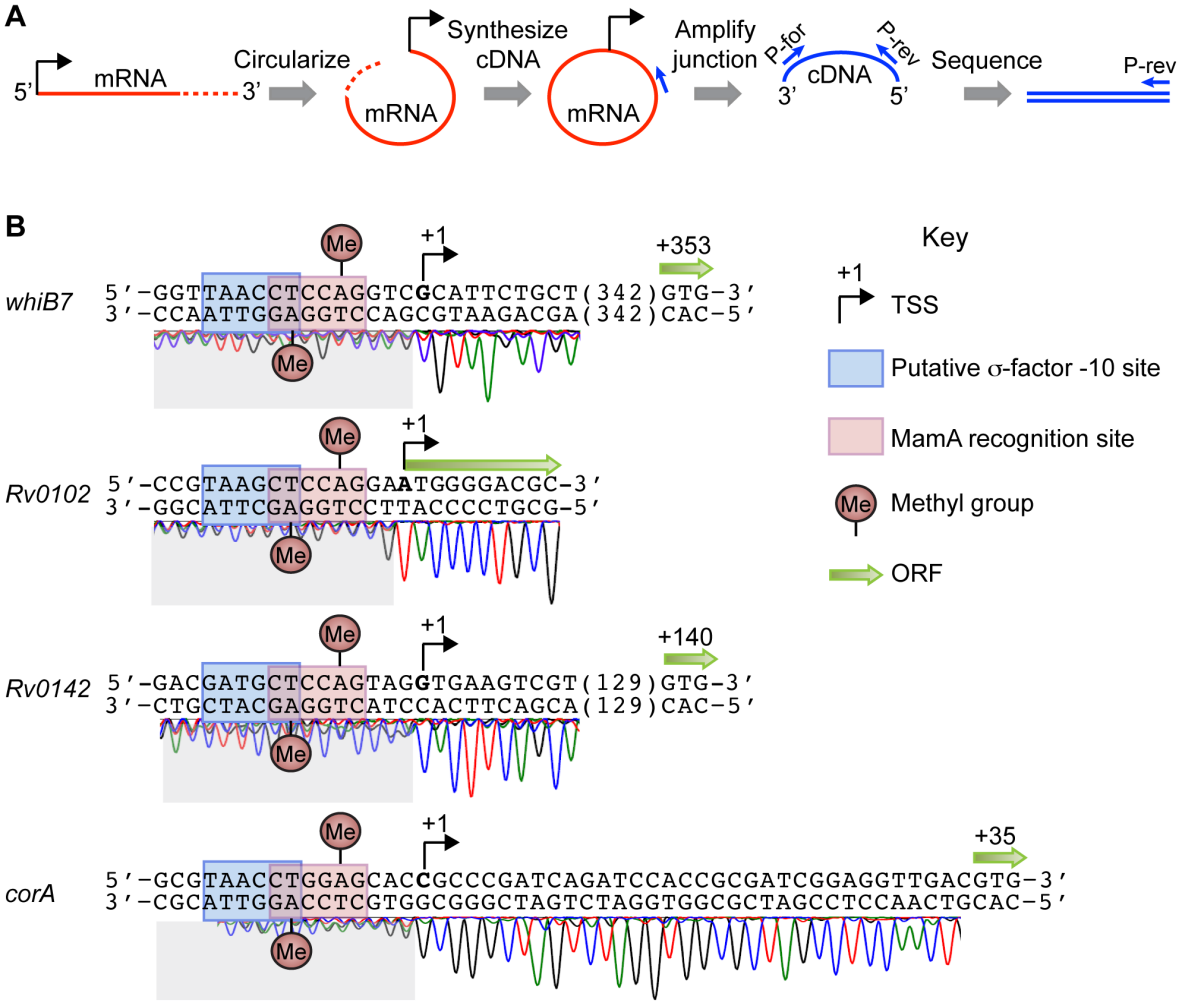
Transcriptional start site (TSS)-mapping reveals a consistent spatial relationship between MamA sites and TSSs of methylation-affected genes. TSSs in strain H37Rv were mapped by the strategy outlined in (A). mRNA was circularized before random-primed synthesis of cDNA. Dashes indicate the variable 3′ end of an mRNA. Gene-specific primers were then used to amplify and sequence 5′-3′ junctions. Junctions appear as transitions from clean to messy sequence due to the variable 3′ ends. (B) TSS-mapping sequence traces are shown for the four genes whose expression is reproducibly affected by MamA. MamA sites, putative sigma factor −10 binding sites, TSSs and ORFs are shown as indicated in the key.

To understand how MamA affects gene expression, we mapped the transcriptional start sites (TSSs) of the qPCR-confirmed genes. We employed a novel strategy based on mRNA circularization in order to map TSSs quickly and accurately (Figure 5A). Total RNA preparations were subject to rRNA depletion and treated with a 5′polyphosphatase to convert 5′ triphosphate “caps” to 5′ monophosphates. The resulting mRNA-enriched samples were circularized by T4 RNA ligase to create molecules containing junctions between 5′ and 3′ ends. Linear cDNA molecules were synthesized from the circular templates by random priming. The 5′-3′ junctions were amplified by gene-specific primers and sequenced by a reverse primer annealing shortly downstream of the start codon. Because the 3′ ends of mRNAs are variable, the 5′-3′ junctions appeared as transitions from monomorphic to polymorphic sequence (Figure 5B). We validated this method by mapping the TSS of *whiB1*, and found that our method predicted a TSS identical to that identified by 5′ Rapid Amplification of cDNA Ends (Figure S3) [56].

The TSSs of *Rv0102*, *Rv0142*, *corA*, and *whiB7* were each located four or five base pairs downstream of a MamA methylation site (Figure 5B). In each case, the predicted sigma factor −10 binding site overlaps with the MamA site such that the last nucleotide of the −10 site is predicted to be methylated on the template strand while the nucleotide three base pairs downstream of the −10 site is predicted to be methylated on the non-template strand (Figure 5B). The conserved spatial relationship between methylation sites and sigma factor binding sites is striking and potentially suggestive of a shared regulatory paradigm among these genes.

### *mamA* deletion reduces survival of *M. tuberculosis* in hypoxia

**Figure 6:**
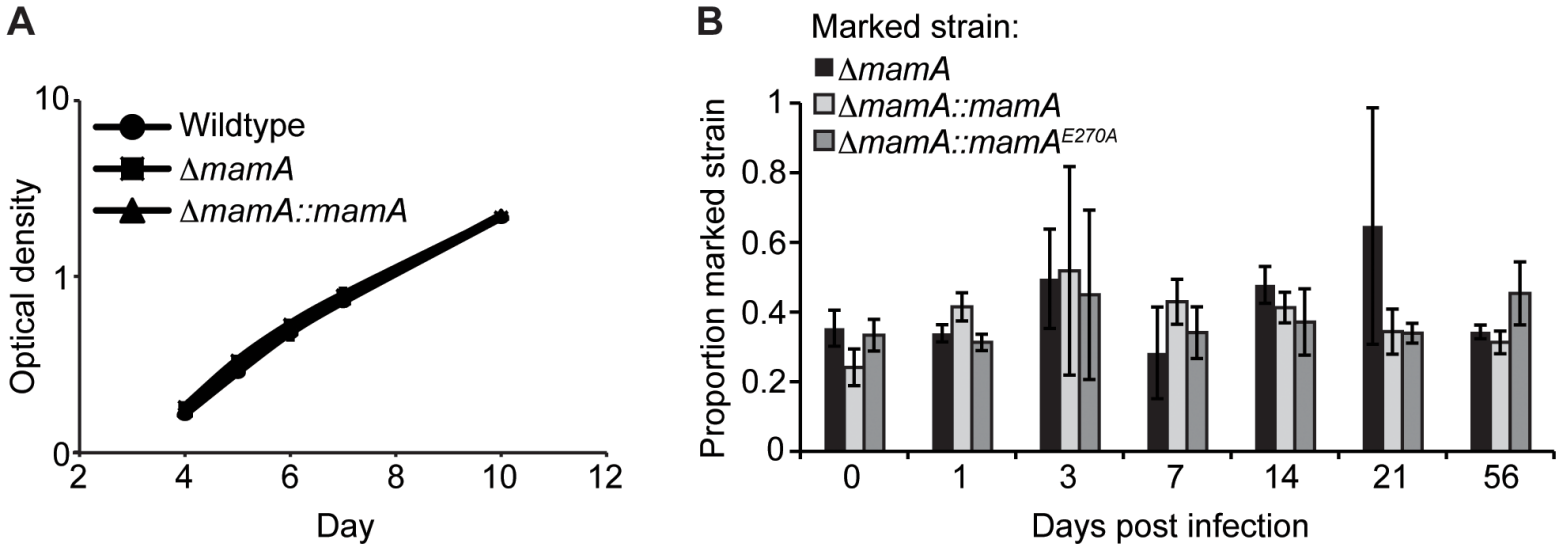
Deletion of *mamA* does not grossly affect growth rate or fitness of *M. tuberculosis* during mouse infection. (A) The indicated H37Rv-derived strains were normalized at a calculated optical density of 0.01 in Sauton's media and monitored by optical density on the days indicated. Points indicate the mean of triplicate cultures and error bars denote standard deviation. Similar results were obtained in 7H9 medium and by plating for CFU. (B) Mice were infected by the aerosol route with approximately 10,000 CFU of a mixture of unmarked wildtype H37Rv and one of three isogenic *mamA* mutants marked with kanamycin resistance. Groups of four mice per condition were sacrificed at the indicated time points and the lung burden of total and marked bacilli was determined. The mean proportion of marked bacteria is indicated. Error bars denote standard deviation.

Given a role for MamA in influencing gene expression, we then sought to determine the functional consequences of losing MamA function. Because DNA methylation plays roles in cell cycle regulation, genome stability and pathogenicity in Proteobacteria [57], [58], we investigated the effects of *mamA* deletion under a number of different conditions. There were no distinguishable differences in growth between wildtype H37Rv, *ΔmamA*, and complemented strains *in vitro* under standard growth conditions (Figure 6A and data not shown). Sensitivity to reactive nitrogen and oxygen species was assessed and no significant differences in survival were observed among the strains (Figures S4A and S4B). *ΔmamA* and complemented strains did not differ in their abilities to compete with wildtype H37Rv in murine infections (Figure 6B and Figure S5). Mutation rates were likewise unaffected by deletion of *mamA*; this was unsurprising given that Mycobacteria lack homologs of the key proteins involved in methyl-directed DNA repair in Proteobacteria (Table S3) [59].

**Figure 7:**
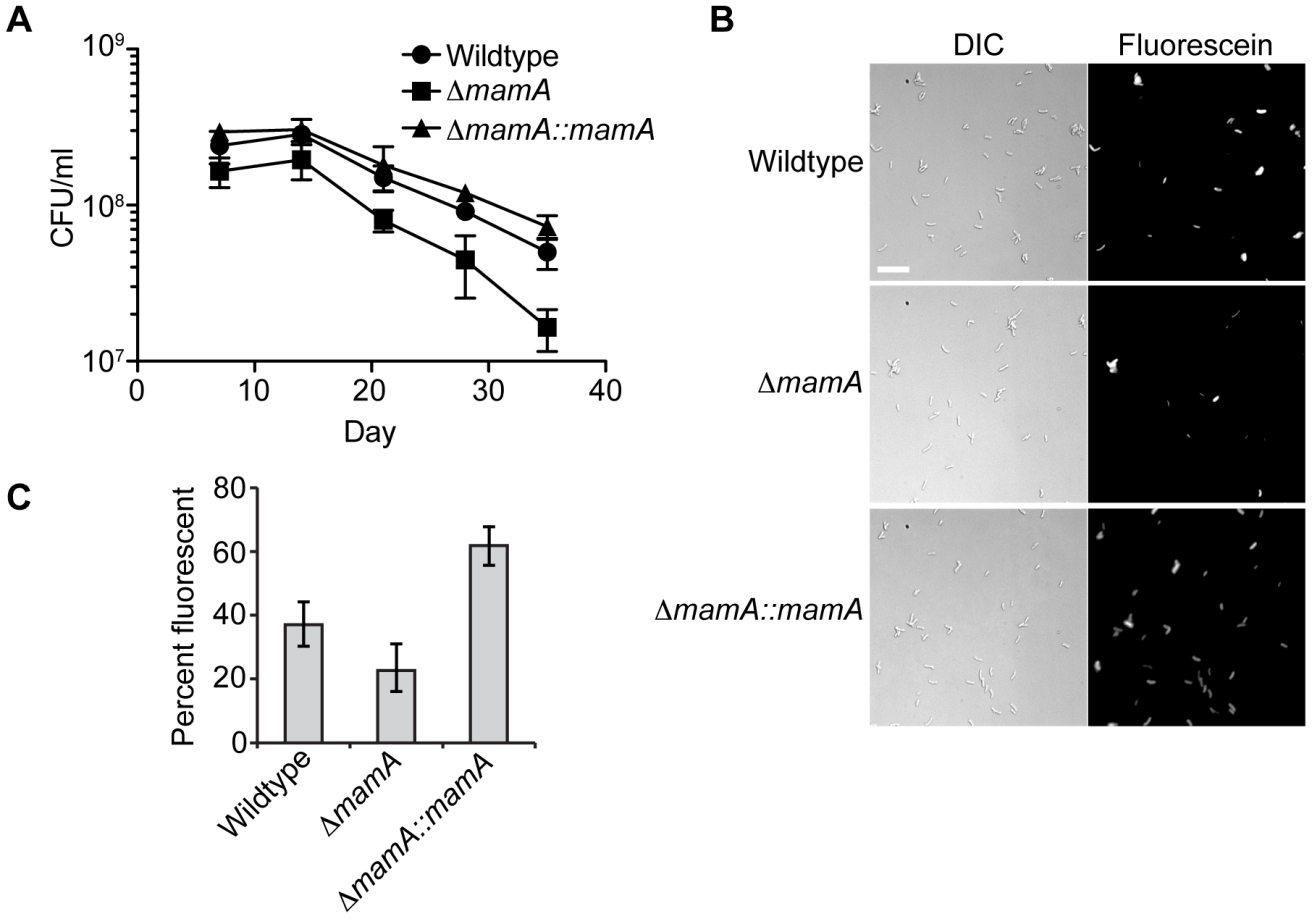
MamA affects viability in hypoxic conditions. The indicated strains of H37Rv were normalized to a calculated density of 3×10^6^ CFU/ml and sealed in bottles containing equal volumes of culture and headspace. (A) Two bottles per strain were opened at the indicated timepoints and CFU/ml determined by plating. Error bars denote standard deviation. The negative slopes of the time points between day 14 and day 35 differ significantly between *ΔmamA* and the other two strains (P<0.05, linear regression of log_10_-transformed values according to the method in [96]). (B) After 28 days, samples of culture were treated with fluorescein diacetate and visualized by microscopy. Only live cells containing active intracellular esterases cleave fluorescein diacetate to produce fluorescent fluorescein. Scale bar = 10 µm. (C) Quantification of percent fluorescent bacteria in three-four fields at day 28. Error bars denote 95% confidence intervals. P<0.05 for all inter-strain comparisons (Fisher's exact test).

Changes in gene expression are often associated with adaptation to new conditions, and we reasoned that MamA might be necessary for adapting to an environment that is not modeled accurately in the mouse. Humans develop hypoxic granulomas that are thought to slow or even arrest the growth of *M. tuberculosis*, but the lesions observed in mice are less organized and remain oxygenated [60]–[62]. Therefore, we therefore tested the ability of wildtype H37Rv, *ΔmamA* and complemented strains survive in hypoxic culture. Bacteria were seeded into vials with a defined headspace/liquid ratio, sealed and incubated with slow stirring, allowing the bacteria to gradually deplete the enclosed oxygen supply and enter a non-replicating state [63]. While all strains displayed reduced viability over time as measured by colony forming units (CFU), the *ΔmamA* strain died at a significantly faster rate than the wildtype parent and the complemented strain (Figure 7A). To assess the viability of the bacteria exposed to hypoxia using a complementary method, a portion of each culture was removed and treated with fluorescein diacetate at day 28. Only viable cells have active intracellular esterases that convert fluorescein diacetate to fluorescein, inducing fluorescence [64]. Consistent with the colony counts, hypoxic cultures of the wildtype and complemented strains had a high proportion of viable cells (∼37–62%), while hypoxic cultures of the *ΔmamA* strain had significantly fewer viable cells (∼23%) (Figure 7B–C).

## Discussion

In this work we demonstrate that *Rv3263* encodes an adenine methyltransferase, MamA, which is responsible for all detectable DNA methylation in the Euro-American lineage strain of *M. tuberculosis*, H37Rv. By deleting *mamA*, we show that in *M. tuberculosis*, adenine methylation alters gene expression. Furthermore, *mamA* is required for optimal bacterial survival in a hypoxic environment. The expression changes mediated by MamA appear subtle in the bulk assays we used. One possible explanation is that methylation may direct greater expression differences in a subpopulation of cells. Single-cell methods will be required to explore this possibility and to determine whether DNA methylation allows heritable (epigenetic) regulation of gene expression in *M. tuberculosis*.

How does MamA alter gene expression? In each case that we have identified, the MamA site overlaps the sigma factor −10 binding site, a promoter region that is directly bound by the RNA polymerase holoenzyme during the initiation of transcription. Strikingly, the MamA sites are located at exactly the same position relative to the −10 sites in the four MamA-affected genes that we examined. This shared spatial configuration contrasts with the locus-specific relationships between Dam methylation sites and promoters at the known methylation-regulated genes in Proteobacteria [5], [65]. The overlap between the promoter MamA sites and the sigma factor −10 binding sites is highly suggestive of a direct effect of methylation on expression of these genes. The lack of broad transcriptional changes or growth rate changes in the *ΔmamA* strain also suggests that the global physiology of the mutant is unperturbed under normal conditions, making indirect effects on transcription less likely. Indeed, the apparently restricted role of MamA under normal growth conditions may have allowed us to detect a category of subtle but direct effects on transcription that might also exist in *E. coli* but which are difficult to detect given the high frequency of Dam sites and dramatic effects of *dam* deletion on cellular physiology.

In the Proteobacteria, methylation has been shown to affect transcription by two broad mechanisms: (1), modulation of repressor binding, and (2), direct modulation of RNA polymerase's interactions with the promoter. Either of these mechanisms could underlie the MamA-dependent expression changes we observe and we propose several potential models for this effect. Methylation could potentially prevent binding of a transcriptional repressor, enhance promoter recognition by the RNA polymerase holoenzyme, increase the melting efficiency of the promoter, or enhance the stability of the open complex.

Three *E. coli* genes are known to be regulated by methylation sites that overlap their sigma factor −10 binding sites, as we observe here, but they do not share a common regulatory paradigm. One of these, *dnaA*, is regulated by repressor binding [6], [8]; another, IS10 transposase, is regulated by direct effects of methylation on RNA polymerase interaction with the promoter [16]; and the third, *glnS*, is Dam-regulated by unknown mechanisms [66]. The *dnaAp2* promoter harbors several Dam sites, one of which overlaps the −10 region. When the Dam sites are hemimethylated following DNA replication a repressor, SeqA, binds and inhibits transcription [6], [8], [11]. Later in the cell cycle, when the promoter becomes fully methylated, expression resumes [12]. This regulatory paradigm clearly falls into the category of methylation-state-dependent repressor binding, which includes other genes with Dam sites in different configurations, such as *pap* and *agn43* [7], [9], [15], [17].

In the case of the IS10 transposase, methylation is thought to alter RNA polymerase interaction with the promoter. Here, methylation directly inhibits expression from the transposase promoter *in vitro*. Hemimethylated promoters have activity that is intermediate between fully methylated and unmethylated promoters [16]. These findings suggest that methylation directly affects either the binding of the RNA polymerase holoenzyme to the promoter, open complex formation, or open complex stability. We note that the effect of methylation on IS10 expression is the opposite of what we observe for MamA-affected genes, and that the methylated bases lie at different positions within the −10 site in the two systems [16].

There are several mechanisms by which DNA methylation may affect open complex formation and stability. DNA is initially melted over a region extending from the second bp of the −10 site to just past the TSS in order to form open complexes [67]. This process involves physical interaction between the −10 site and the 2.4 region of sigma as well as a more recently identified interaction between the DNA shortly downstream of the −10 site and the 1.2 region of sigma [68]–[71]. N^6^-MdA can reduce the melting temperature of DNA heteroduplexes *in vitro* [72], which may make open complex formation more thermodynamically favorable. DNA melting efficiency is thought to be important in open complex formation in part because of the AT-rich nature of −10 sites in most bacteria, and because a GC-rich region between the −10 site and TSS is important for stringent control of some promoters [70], [73], [74]. In MamA-affected genes, N^6^-MdA in the −10 site and between the −10 site and TSS could therefore potentially enhance open complex formation and consequently increase expression.

It is also possible that region 1.2 of sigma could make direct contact with the N^6^-MdA located between the −10 site and TSS on the non-template strand, and that such an interaction could increase open complex stability directly. Region 1.2 of sigma was shown in *E. coli* to make direct contact with bases on the non-template strand between the −10 site and TSS of *rrnB* P1 and λP_R_ [70], [75]. In *Bacillus subtilis*, changing the base at position −5 of *rrnB* P1 from T to A resulted in a decrease in sensitivity of the promoter to GTP levels, indicating that the stability of its open complex was increased [76]. Together, these data suggest that the sequence of the region between the −10 site and the TSS matters for reasons beyond GC content, and the non-template strand in particular plays an important role. *In vitro* studies will be required to elucidate the effects of MamA-mediated methylation on interactions between *M. tuberculosis* promoters and RNA polymerase holoenzyme.

The MamA-affected genes are not obviously related with respect to pathway or function, although several are involved in stress responses. *Rv0102* is an essential gene predicted to encode an integral membrane protein of unknown function [77]. It is not reported to undergo major transcriptional changes [78], although it may be modestly induced by oxidative stress [79]. *Rv0142* is predicted to encode a DNA glycosylase and is strongly induced in response to oxidative stress in a σ^H^-dependent fashion [79]–[81]. It may also be induced by nitrosative stress [79]. *CorA* encodes a predicted magnesium and cobalt transporter that may be modestly induced by thioridazine, proton gradient disrupters, and oxidative stress [78], [79], [82]. We find that induction of *Rv0142* and *corA* in response to oxidative stress appears to occur normally in a *ΔmamA* strain (data not shown), suggesting that MamA methylation affects the basal transcription of these genes but not the higher-level transcription that occurs during oxidative stress. *WhiB7* is a transcriptional regulator that is induced by stationary phase, multiple antibiotics, heat shock, and iron starvation; disruption of *whiB7* leads to increased antibiotic susceptibility [83], [84]. Further work is needed to understand whether the hypoxia-survival defect in *ΔmamA* is related to the gene expression differences we detected or is a result of other effects of *mamA* deletion on hypoxia-specific gene expression. Gene expression profiling of 2-week-old hypoxic cultures suggested that a small number of genes are differentially expressed in the absence of *mamA*, similar to the extent of gene expression changes under standard *in vitro* growth conditions. These include some genes influenced by MamA in aerobic growth and some novel potential targets (data not shown). Further studies are required to validate these findings and understand their implications for *M. tuberculosis* survival under hypoxic conditions.

Interestingly, MamA is partially inactivated by a point mutation in most strains of the Beijing lineage of *M. tuberculosis*, while a second methyltransferase, HsdM, is active in the Beijing lineage and inactivated by a point mutation in most Euro-American strains. Non-synonymous methyltransferase mutations are found in other *M. tuberculosis* lineages as well (TB Database, [43]), although the effects of these mutations on methyltransferase activity are unknown. A few of the oldest strains of the Beijing and Euro-American lineages, as well as strains from the rim of the Indian Ocean, appear to encode intact copies of both MamA and HsdM. Further work will be needed to understand how the roles of DNA methylation may differ according to genetic background. It is possible that MamA is important for fitness of Euro-American strains during infection-associated hypoxia, but is unnecessary or even detrimental in modern Beijing lineage strains due to their altered hypoxia-response gene regulatory networks [39], [40]. Loss of MamA function may affect other aspects of the Beijing lineage strains. The insertion sequence IS*6110* has a MamA site that overlaps both the inverted repeat and the presumed promoter for the transposase gene. The number of IS*6110* elements is higher in Beijing lineage strains than in other lineages, suggesting that transposition may be more frequent in Beijing lineage strains [85]. IS*6110* activity may be beneficial because it introduces genetic variability into a clonal species that lacks opportunities for horizontal gene transfer [86]. In *E. coli* IS10 transposition is altered by Dam through both expression-dependent and –independent mechanisms [16]. Although we did not detect changes in IS*6110* transposase expression in strains lacking *mamA* (data not shown), future studies may indicate an effect of MamA on IS*6110* transposition rates.

In this work we report the first investigation of the functional effects of DNA methylation in *M. tuberculosis*, as well as basic characterization of where and how DNA methylation occurs in this globally important bacterium. Methylation enhances expression of several genes that have methylation sites located in identical positions within their promoters, consistent with a shared regulatory paradigm. The activity of the methyltransferase MamA is required for normal survival of hypoxia, indicating that it is likely an important mediator of adaptation to this physiologically relevant stressor. Different methyltransferases predominate in different lineages of *M. tuberculosis*, suggesting that methylation-mediated regulatory pathways may contribute to lineage-specific characteristics.

## Materials and Methods

### Ethics statement

Animal experiments were performed in strict accordance with the National Institutes of Health guidelines for housing and care of laboratory animals, with institutional regulations after protocol review, and with approval by the Harvard Medical Area Standing Committee on Animals. The animal protocol was approved by the Harvard University IACUC (protocol number 03000).

### Strain construction and culture conditions

*M. tuberculosis* strains were grown in Middlebrook 7H9 or 7H10 media supplemented with 10% OADC (Oleic Albumin Dextrose Catalase, Becton Dickinson), glycerol, and 0.05% Tween 80 unless otherwise specified. H37Rv strains were derived from the ATCC lineage. Unmarked *mamA* (*Rv3263*) and *hsdM* deletion strains were constructed by a two-step process. Plasmids pSS002 and pSS004 were derived from pJM1 [87] and contained 1 kb of the sequence upstream and downstream of *hsdM* and *mamA*, respectively, with 24–27 base pairs of coding sequence and a stop codon in between the two flanks. Plasmids were linearized with restriction enzymes cutting within one of the flanks before transformation into H37Rv. Integrants were selected with 50 µg/ml hygromycin. Counterselection with 7% sucrose was followed by PCR screening to identify isolates that subsequently underwent second crossovers resulting in loss of the plasmid and *hsdM* or *mamA* coding sequences. Complementation vectors were derived from pJEB402, which integrates as a single copy into the L5 *attB* site [88]. The *mamA* coding sequence and 33 upstream bases (assumed to contain the RBS) were cloned behind the MOP promoter present in pJEB402, creating plasmid pSS030. We performed PCR with primers containing a central mutation in order to change nt 809 of the coding sequence from “A” to “C” creating pSS040. An equivalent strategy was used to insert the active site mutations N127D and Y130A into plasmids pSS075 and pSS077, respectively. For complementation with wildtype *hsdM*, the *hsdM* coding sequence and 23 upstream nt assumed to contain the RBS were cloned behind the MOP promoter in pJEB402 to produce plasmid pSS079.

### Genomic DNA isolation and Southern blotting

Cultures were grown to an optical density of between 0.7 and 1.1 unless otherwise specified. Cell pellets were inactivated by chloroform-methanol (ratio 2∶1), pelleted, resuspended in 0.1M Tris and 1 mM EDTA, pH 8–9, and lysed with lysozyme overnight at a final concentration of 100 µg/mL. Lysates were treated with 1% SDS and 100 µg/mL proteinase K (IBI Scientific) (final concentrations) for 3 hours at 50°C followed by phenol-chloroform extraction according to standard procedures, RNase (MO BIO) treatment (25 µg/mL for 1 hour at 37°C), and a second phenol-chloroform extraction. Two µg DNA was digested with PvuII (NEB) for Southern blotting. Blotting was performed according to standard protocols. DIG-labeled probe was made and detected with Roche DIG DNA Labeling and Detection Kit and Roche DIG Wash and Block Buffer Set according to the manufacturer's instructions.

### Plasmid isolation and sequence trace comparison

Plasmids for sequence trace comparison were constructed by digesting pMV762 [89] with BamHI and HindIII (NEB) and ligating in an annealed oligonucleotide duplex containing the sequence of interest (see Table S1 for oligonucleotide sequences). pSS012 contains the full 10 base pair sequence and other variants are listed in Table S1. pMV762 contains multiple complete and partial MamA sites, and these were sequenced as well. Plasmids were isolated from *M. tuberculosis* using a variation of a published protocol [90]. Briefly, 30 ml of culture was pelleted and inactivated by overnight incubation with a 4∶1 ratio of chloroform∶methanol at 4°C. After centrifugation and removal of the liquid phases, pellets were resuspended in 200 µl of lysozyme solution [90] and incubated 4–18 h at 4°C. 400 µl of alkaline SDS solution [90] was added and samples incubated 30 min at 4°C with agitation. Buffer N3 (700 µl) from a Qiagen miniprep kit was added and samples centrifuged at maximum speed for 10 min. The supernatant was then applied to a Qiagen miniprep spin column and sample was processed according to the manufacturer's instructions. *E. coli* derived plasmids were propagated in both DH5-alpha and a *dam dcm* deletion strain (NEB). No differences were observed between sequence traces from the two *E. coli* strains.

### Mass spectrometry

Five µg of DNA was digested to nucleosides enzymatically with the addition of deaminases to reduce artifactual deamination due to contaminating deaminases in commercial enzyme preparations [91]. Isotopically labeled internal standards for 5-MdC and N^6^-MdA were synthesized and spiked into the digestion reactions (EGP manuscript in preparation and [92]). An HPLC method that separates all four methylated products and the canonical nucleosides was developed utilizing a Cogent Diamond Hydride aqueous normal phase column (2.1×250 mm, 4 µm particle, 100 Å pore size; Microsolv Technology Corporation, Eatontown, NJ) with an isocratic step gradient of 0.1% acetic acid in acetonitrile/water (EGP manuscript in preparation). The LC-MS/MS analysis was performed on an Agilent 1100 HPLC coupled to an AB Sciex API 3000 triple quadrupole mass spectrometer in positive ion multiple reaction monitoring mode utilizing only 50–200 ng of DNA. Transitions monitored were *m/z* 266-150 (N^6^MdA), *m/z* 271-154 ([^15^N_5_]^6^-MdA), *m/z* 242-126 (5-MdC/N^4^MdC), *m/z* 254-133 ([^13^C_9_^15^N_3_]-5-MdC), *m/z* 258-142 (5-hydroxymethylcytidine), *m/z* 252-136 (dA), *m/z* 243-127 (dT), *m/z* 228-112 (dC), and *m/z* 268-152 (dG). For each case, the monitored transition represents the loss of the 2′-deoxyribose. The areas under the curve of each nucleoside transition were quantitated and compared to calibration curves (r = 0.99). There were three biological replicates and at least two technical replicates per sample.

### Murine infections

Female C57BL/6 mice were purchased from Jackson Laboratory (Bar Harbor, ME). Freshly grown cultures of wildtype H37Rv (kanamycin sensitive), *ΔmamA*::pJEB402, *ΔmamA*::pSS030, and *ΔmamA*::pSS040 (kanamycin resistant) were washed and cell densities were estimated by optical density. Equal quantities of wildtype bacteria were mixed with each of the three marked strains in order to perform three separate competition experiments. Mice were infected by the aerosol route with approximately 10^4^ CFU. Four mice per group were sacrificed at the indicated time points and bacterial burden in the lung and spleen were determined by plating homogenized organs on plates both with and without 25 µg/ml kanamycin. Animal experiments were performed in accordance with the National Institutes of Health guidelines for housing and care of laboratory animals, with institutional regulations after protocol review, and with approval by the Harvard Medical Area Standing Committee on Animals.

### RNA isolation and cDNA synthesis

RNA was isolated from cultures grown to OD 0.8–0.9 in the absence of antibiotics. Twenty ml of culture was added to 20 ml of RNAlater (Ambion) and incubated for 10 min. Ten ml of water was added immediately before centrifugation for 15 min. Pellets were resuspended in one ml Trizol (Invitrogen) and subject to bead-beating for 45 s and 30 s in a FastPrep-24 instrument (MP) before continuing according to the manufacturer's instructions. RNA samples were then treated with 10 U DNase Turbo (Ambion) for 1 h and purified with an RNeasy kit (Qiagen) according to the manufacturer's instructions, with the addition of RNaseOUT (Invitrogen) to the water used for elution. For quantitative PCR and TSS mapping, cDNA was synthesized as follows. One µg of RNA was mixed with 1.3 µl of 3 mg/ml random hexamers (Invitrogen), denatured at 70°C for 10 min and snap-cooled on ice before adding 4 µl 5X Superscript First Strand Buffer, 1 µl of dNTPs at 10 mM each, 0.4 µl of 500 mM DTT, 1 µl RNaseOUT, and 1 µl Superscript III (Invitrogen). Reactions were performed overnight at 42°C. RNA was degraded with the addition of 10 µl each 500 mM EDTA and 1 N NaOH and heating to 65°C for 15 min, followed by neutralization with 25 µl of 1M Tris pH 7.5. cDNA was then purified over Qiagen MinElute columns according to the manufacturer's instructions.

### Quantitative PCR (qPCR)

qPCR primers are listed in Table S4. Each 20 µl reaction contained 100–200 pg of cDNA, 2.5 pg of each primer, and 10 µl of iTaq SYBR Green Supermix with ROX (Biorad). Reactions were run in an Applied Biosystems 7300 Real Time PCR System with the following program: 50°C/2 min, 95°C/5 min, and 40 cycles of 95°C/15 s and 61°C/30 s. Expression values normalized to *sigA* were calculated by the Δct method. Expression differences were compared by ANOVA with Tukey's post-test using GraphPad Prism 5. qPCR was performed on separate biological replicates from those used for microarray analysis.

### Microarray analysis

RNA was extracted from triplicate cultures of indicated strains as described above for expression analysis with the Affymetrix custom-designed GeneChip MTbH37Rva520730F for *M. tuberculosis* (GEO platform number GPL17082, designed at the Broad Institute). Microarrays were run by the Boston University Microarray Core, who prepared the probes, hybridized and scanned the arrays according to the manufacturer's directions for prokaryotic samples with high GC content. Expression estimates were derived from probe-level hybridization intensities using RMA [93] in Expression Console (Affymetrix). Differential expression of non-intergenic features was assessed using 1-way ANOVA and for each ANOVA p-value we calculated a False Discovery Rate (FDR) using the method of Benjamini and Hochberg [94] to account for the large number of genomic features we interrogated. The ANOVA and FDR calculations were done using version 2.14 of the R Language for Statistical Computing [95]. Data are available on GEO, accession number GSE46432.

### Transcriptional start site (TSS) mapping

Total RNA samples were subject to two consecutive rounds of rRNA depletion with the MICROBExpress kit (Ambion) according to the manufacturer's instructions. To convert the natural 5′ triphosphates of mRNAs to 5′ monophosphates, approximately one µg of enriched mRNA was treated with 5′Polyphosphatase (Epicentre) for 30 minutes at 37°C in a 10 µl reaction containing 1 µl of enzyme and the supplied buffer, followed by RNeasy purification (Qiagen). The resulting sample was then circularized in 50 µl reactions contained 200 ng of RNA, 2 µl of T4 RNA ligase I (Epicentre), and ATP and buffer according to the manufacturer's recommendations in a final volume of 50 µl. Reactions were allowed to proceed for 2 h at 37°C and purified with RNeasy. cDNA was synthesized as described. Primer sets for genes of interest were designed such that the forward primer annealed approximately 100 base pairs upstream of the stop codon and the reverse primer annealed approximately 150–200 base pairs downstream of the start codon (Table S5). PCR reactions were in 25 µl volumes and contained 0.25 µl of Phusion polymerase (Finnymes), 1.25 pg of each primer, 0.2 µl of 25 mM each dNTPs, 1X GC buffer (Finnzymes), and 12–15 ng of cDNA. Reactions were performed with genomic DNA and with cDNA derived from non-circularized RNA for comparison. Cycler conditions were 98°C/2 min, 30 cycles of 98°C/15 s, 60°C/15 s, 72°C/15 s, and a final extension of 72°C/5 min. Entire reactions were then run on a 1% agarose gel. Bands present in reactions templated from circularized samples but absent in reactions templated from non-circularized samples or genomic DNA were excised and purified with Qiagen spin columns. One or two bands were identified for each gene, and some were sharp and distinct while others appeared as smears. Entire gel-extracted products were then concentrated under vacuum and subjected to a second round of PCR with the same primers, scaled up to 50 µl and with the addition of 1 µl of DMSO. Entire reactions were again run on a gel and the purified products were sequenced directly with their respective PCR primers.

### Hypoxia survival experiments

The indicated strains of H37Rv were grown in 7H9 media supplemented with ADC and Tween-80 containing selective antibiotics if necessary. Seed cultures were washed twice, normalized and inoculated at a calculated density of 3×10^6^ cfu/ml into 31 ml fresh media without antibiotics in a rubber stopper-sealed serum bottle (62 ml total volume). Cultures were shaken at ∼120 rpm at 37°C with an intermittent manual homogenization in case of cell precipitation. Two bottles per strain were opened at the indicated time points and cfu/ml was determined after serial dilutions and plating onto 7H10 agar supplemented with OADC.

At 28 days, each hypoxic culture used for CFU determination was also was stained with fluorescein diacetate. 10 ml of culture was pelleted and resuspended in 2 ml of PBS with 0.05% Tween-80. Fluorescein diacetate was prepared as a 100X stock in acetonitrile and methanol (1∶1) and added to resuspended cells to a final concentration of 50 µg/ml. After 30 minutes of incubation at 37°C, the dye-treated cells were washed with PBS-tween to remove the residual dye and then fixed with formalin. Fluorescent cells were visualized microscopically (DeltaVison, AppliedPrecision Inc.) using identical exposure settings for all strains. Death rates were calculated by linear regression analysis of log_10_-transformed data for time points between day 14 and day 35 (inclusive) in GraphPad Prism 5, which compares the significance of differences in slope using the method described in [96].

## Supporting Information

Figure S1 **Expression of** ***mamA*** **in** ***M. tuberculosis*** **H37Rv.** Expression of *mamA* relative to *sigA* was measured by qPCR. Expression of *mamA* was not detectable in the *ΔmamA* strain. The complemented strain displays approximately 3.5-fold more *mamA* expression than the wildtype strain, likely because the complementation vector contains a Mycobacterial optimized promoter (MOP) in place of the native promoter. Error bars denote standard deviation of mean of technical triplicates.
(PDF)

Figure S2 **Quantification of sequence trace comparisons.** The area under the curve (AUC) was determined for each peak in each of the sequence traces displayed in Figure 2A (Adobe Photoshop CS5) and normalized to the mean AUC for that trace. The percent difference in AUC in traces from plasmid isolated from *M. tuberculosis* strains compared to *E. coli* was determined for each peak. Gray shading indicates the mean percent difference plus two standard deviations; differences that exceeded this threshold were considered to be significant. (A) Quantification of “Top Strand” traces shown in Figure 2A. (B) Quantification of “Bottom Strand” traces shown in Figure 2A. Note that the peaks for nucleotides C4 and C5 overlapped substantially and were therefore analyzed together.
(PDF)

Figure S3 **Confirmation of the** ***whiB1*** **TSS.** The TSS of *whiB1* was mapped as in Figure 5. TSS is indicated by the black arrow.
(PDF)

Figure S4 **Deletion of** ***mamA*** **does not affect sensitivity to nitrosative or oxidative stress in strain H37Rv.** (A) Log-phase cultures were exposed to 10 mM DETA-NO for 24 and then plated for CFUs to assess survival compared to untreated cultures. Mean percent survival of triplicate cultures is shown. Error bars denote standard deviation. Differences between strains are not significant (t-test). Data are representative of two independent experiments. (B) Late log-phase cultures were pelleted and resuspended to OD 0.2 in catalase-free media with the addition of H_2_O_2_ to the indicated final concentrations. Growth over the next four days was monitored by OD. Mean ODs of triplicate cultures are shown. Error bars are omitted for the sake of clarity.
(PDF)

Figure S5 **MamA status does not affect growth of H37Rv in mice.** Mice were infected by the aerosol route with approximately 10,000 CFU of a mixture of unmarked wildtype H37Rv and one of three isogenic *mamA* mutants marked with kanamycin resistance. Groups of four mice per condition were sacrificed at the indicated time points and the lung burden of total and marked bacilli was determined. The mean CFU on 7H10 plates without drug (total *M. tuberculosis*), with kanamycin (mutant strain), and the calculated difference (wildtype H37Rv) are shown. Error bars denote standard deviation. (A) Infection with a mixture of unmarked H37Rv and kan^R^ H37Rv *ΔmamA*. (B) Infection with a mixture of unmarked H37Rv and kan^R^ H37Rv *ΔmamA::mamA*. (C) Infection with a mixture of unmarked H37Rv and kan^R^ H37Rv *ΔmamA::mamA^E270A^*. Data are from the same experiment as shown in Figure 6B.
(PDF)

Figure S6 **Structures of methylated 2′deoxynucleosides examined in this study.** Figure made in ChemDraw.
(PDF)

Methods S1 **Materials and methods for experiments shown in figures S1, S2, S3, S4, S5.**
(DOCX)

Table S1 **Putative MamA recognition sequences tested by sequence trace comparison.**
(DOCX)

Table S2 **Complete aerobic microarray dataset.**
(XLSX)

Table S3 **Mutation rates in wildtype and** ***ΔmamA*** **strains.**
(DOCX)

Table S4 **Primers used for quantitative PCR.**
(DOCX)

Table S5 **Primers used for transcriptional start site mapping.**
(DOCX)
