# Supplementary figures and images for: DNA Methylation Impacts Gene Expression and Ensures Hypoxic Survival of Mycobacterium tuberculosis
Source: PLoS Pathog. 2013 Jul 4;9(7):e1003419. doi: 10.1371/journal.ppat.1003419 (PMC3701705; doi:10.1371/journal.ppat.1003419)

Figure S1

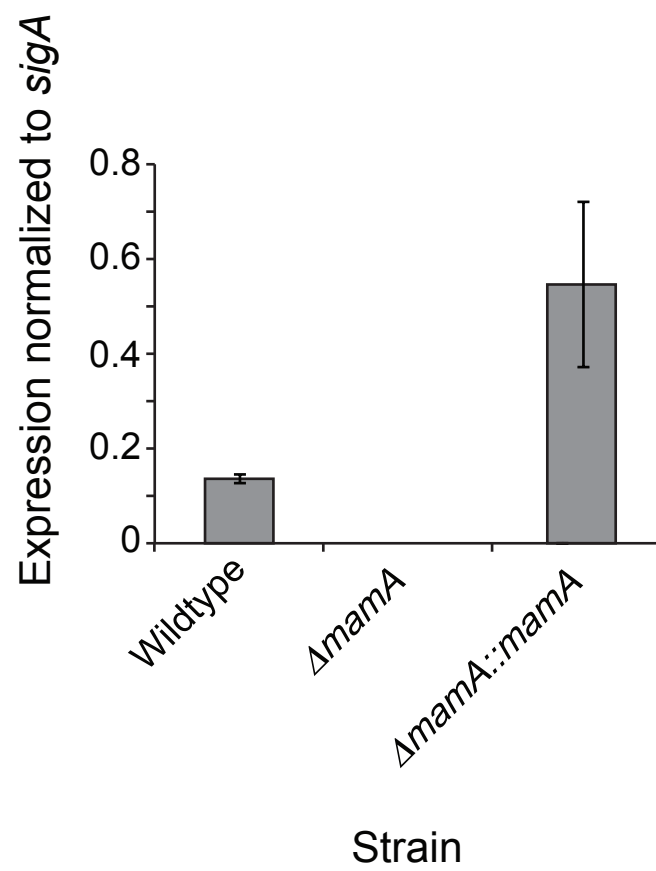

Supplement: Figure S1 — Expression of mamA in M. tuberculosis H37Rv. Expression of mamA relative to sigA was measured by qPCR. Expression of mamA was not detectable in the ΔmamA strain. The complemented strain displays approximately 3.5-fold more mamA expression than the wildtype strain, likely because the complementation vector contains a Mycobacterial optimized promoter (MOP) in place of the native promoter. Error bars denote standard deviation of mean of technical triplicates. (PDF) [file ppat.1003419.s001.pdf]

Figure S2

A

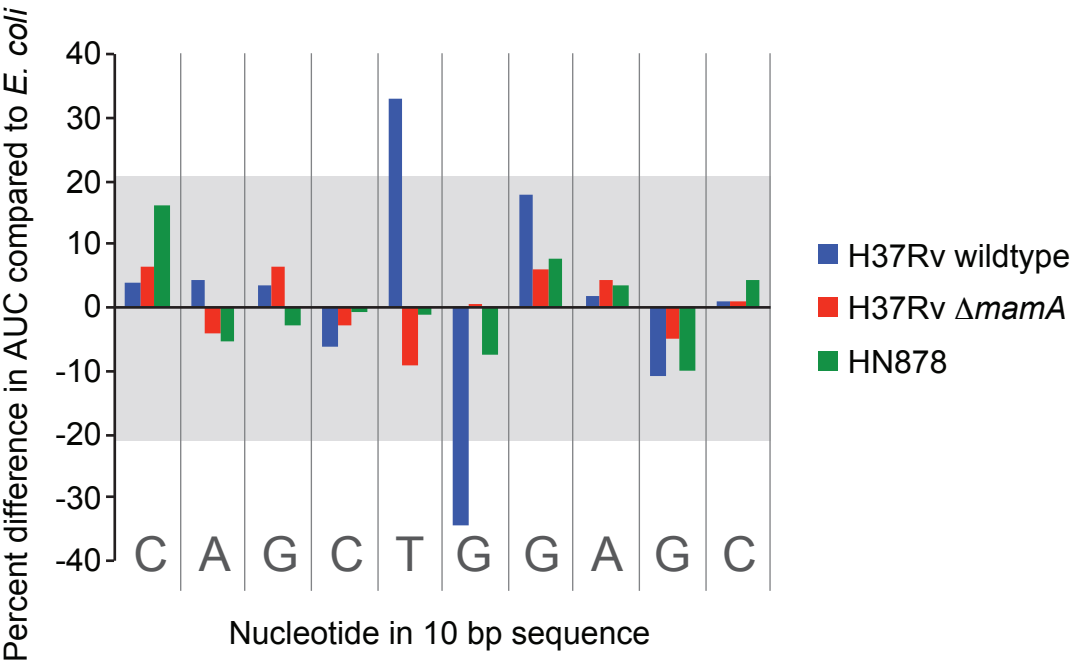

B

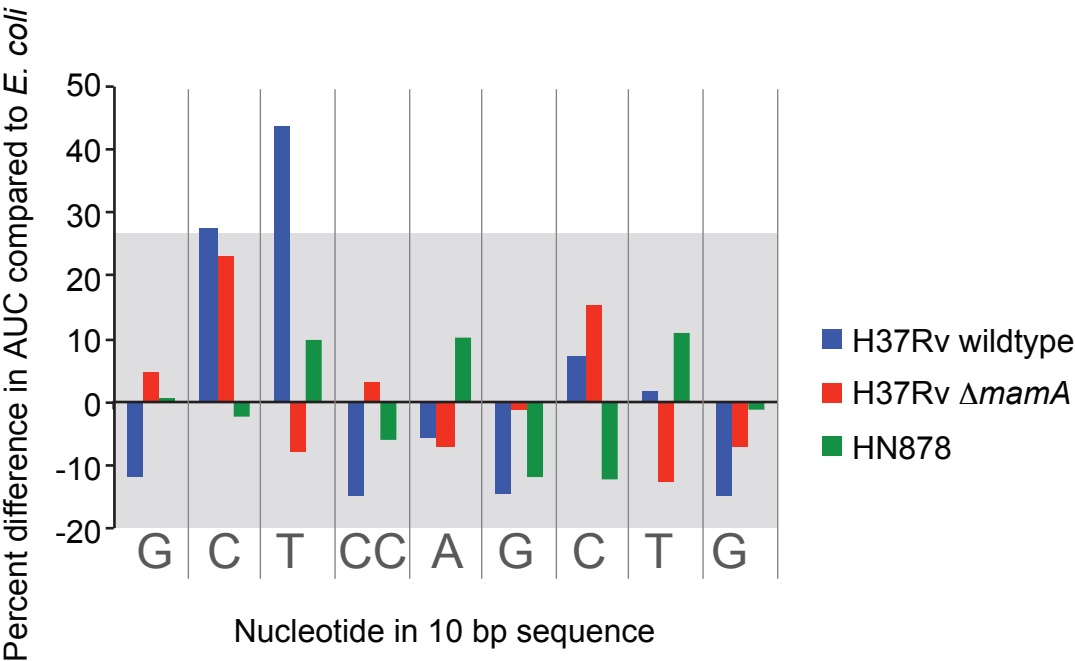

Supplement: Figure S2 — Quantification of sequence trace comparisons. The area under the curve (AUC) was determined for each peak in each of the sequence traces displayed in Figure 2A (Adobe Photoshop CS5) and normalized to the mean AUC for that trace. The percent difference in AUC in traces from plasmid isolated from M. tuberculosis strains compared to E. coli was determined for each peak. Gray shading indicates the mean percent difference plus two standard deviations; differences that exceeded this threshold were considered to be significant. (A) Quantification of “Top Strand” traces shown in Figure 2A. (B) Quantification of “Bottom Strand” traces shown in Figure 2A. Note that the peaks for nucleotides C4 and C5 overlapped substantially and were therefore analyzed together. (PDF) [file ppat.1003419.s002.pdf]

Figure S3

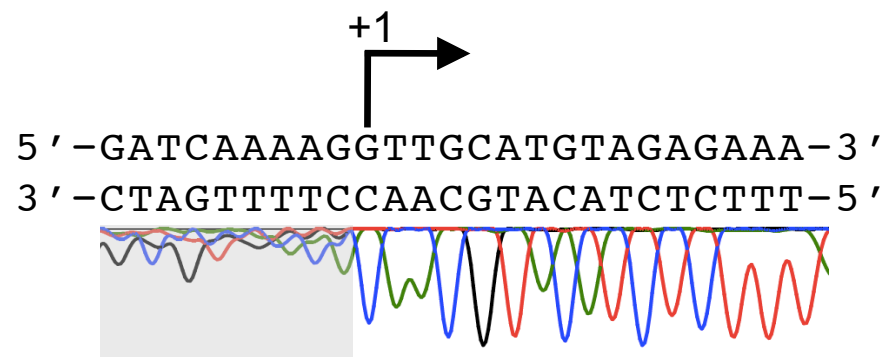

Supplement: Figure S3 — Confirmation of the whiB1 TSS. The TSS of whiB1 was mapped as in Figure 5. TSS is indicated by the black arrow. (PDF) [file ppat.1003419.s003.pdf]

Figure S4

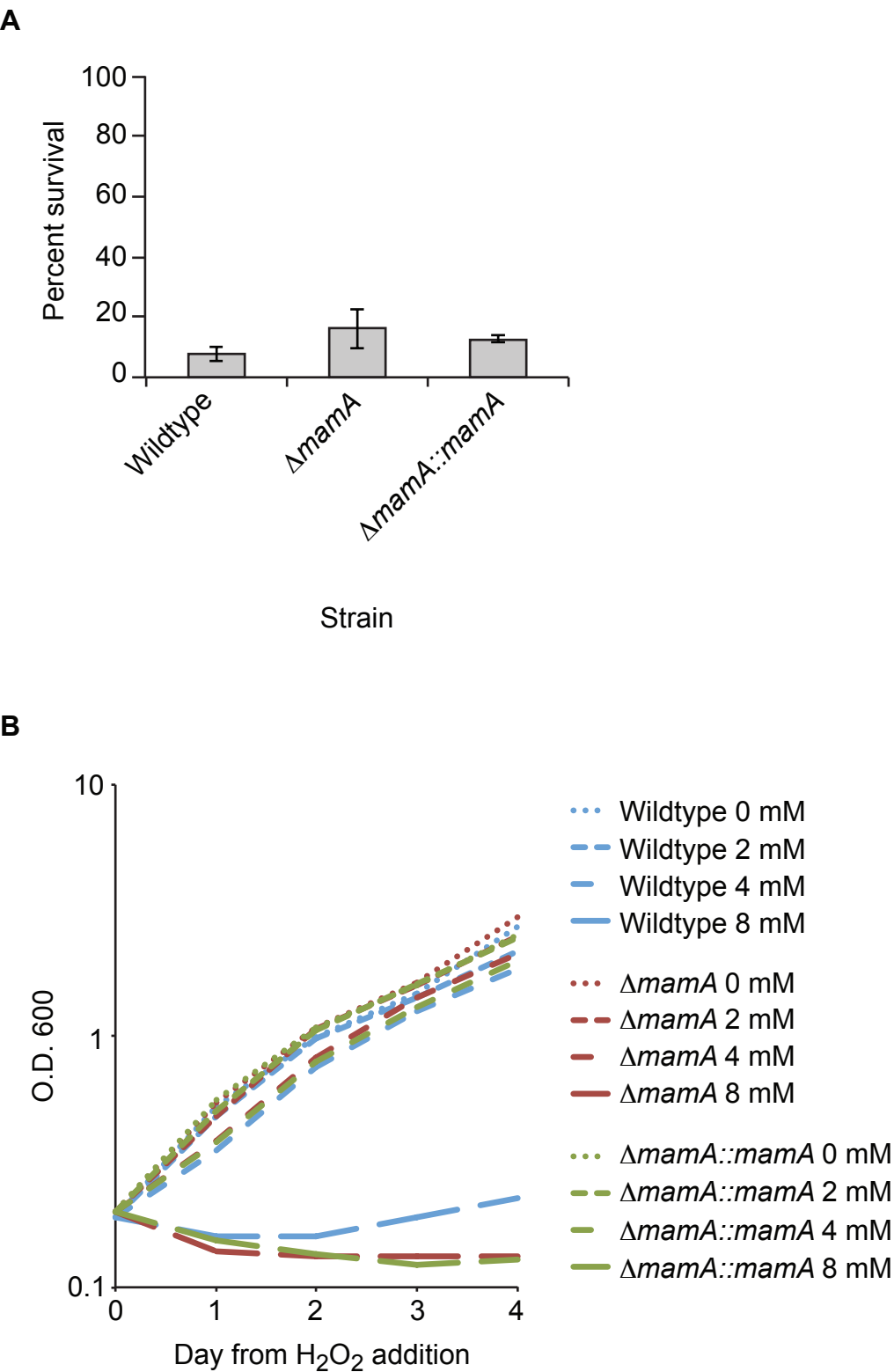

Supplement: Figure S4 — Deletion of mamA does not affect sensitivity to nitrosative or oxidative stress in strain H37Rv. (A) Log-phase cultures were exposed to 10 mM DETA-NO for 24 and then plated for CFUs to assess survival compared to untreated cultures. Mean percent survival of triplicate cultures is shown. Error bars denote standard deviation. Differences between strains are not significant (t-test). Data are representative of two independent experiments. (B) Late log-phase cultures were pelleted and resuspended to OD 0.2 in catalase-free media with the addition of H2O2 to the indicated final concentrations. Growth over the next four days was monitored by OD. Mean ODs of triplicate cultures are shown. Error bars are omitted for the sake of clarity. (PDF) [file ppat.1003419.s004.pdf]

Figure S6

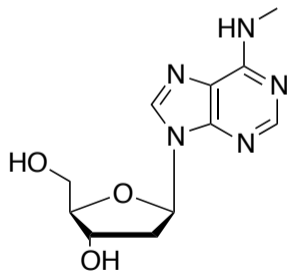

N<sup>6</sup>-MethylidA

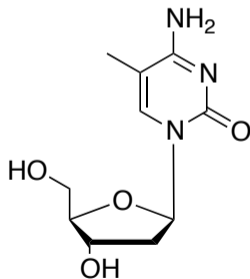

5-MethylidC

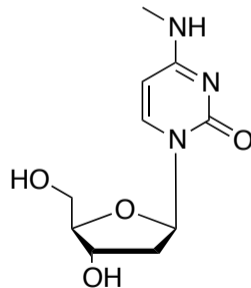

N<sup>4</sup>-MethylidC

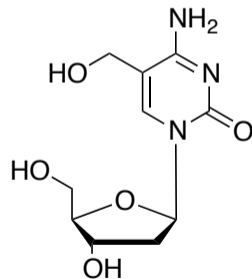

5-HMdC

Supplement: Figure S6 — Structures of methylated 2′deoxynucleosides examined in this study. Figure made in ChemDraw. (PDF) [file ppat.1003419.s006.pdf]
