# Supplementary material for: DNA Methylation Impacts Gene Expression and Ensures Hypoxic Survival of Mycobacterium tuberculosis
Source: PLoS Pathog. 2013 Jul 4;9(7):e1003419. doi: 10.1371/journal.ppat.1003419 (PMC3701705; doi:10.1371/journal.ppat.1003419)
Supplement: Figure S5 — MamA status does not affect growth of H37Rv in mice. Mice were infected by the aerosol route with approximately 10,000 CFU of a mixture of unmarked wildtype H37Rv and one of three isogenic mamA mutants marked with kanamycin resistance. Groups of four mice per condition were sacrificed at the indicated time points and the lung burden of total and marked bacilli was determined. The mean CFU on 7H10 plates without drug (total M. tuberculosis), with kanamycin (mutant strain), and the calculated difference (wildtype H37Rv) are shown. Error bars denote standard deviation. (A) Infection with a mixture of unmarked H37Rv and kanR H37Rv ΔmamA. (B) Infection with a mixture of unmarked H37Rv and kanR H37Rv ΔmamA::mamA. (C) Infection with a mixture of unmarked H37Rv and kanR H37Rv ΔmamA::mamAE270A. Data are from the same experiment as shown in Figure 6B. (PDF) [file ppat.1003419.s005.pdf]

Figure S5

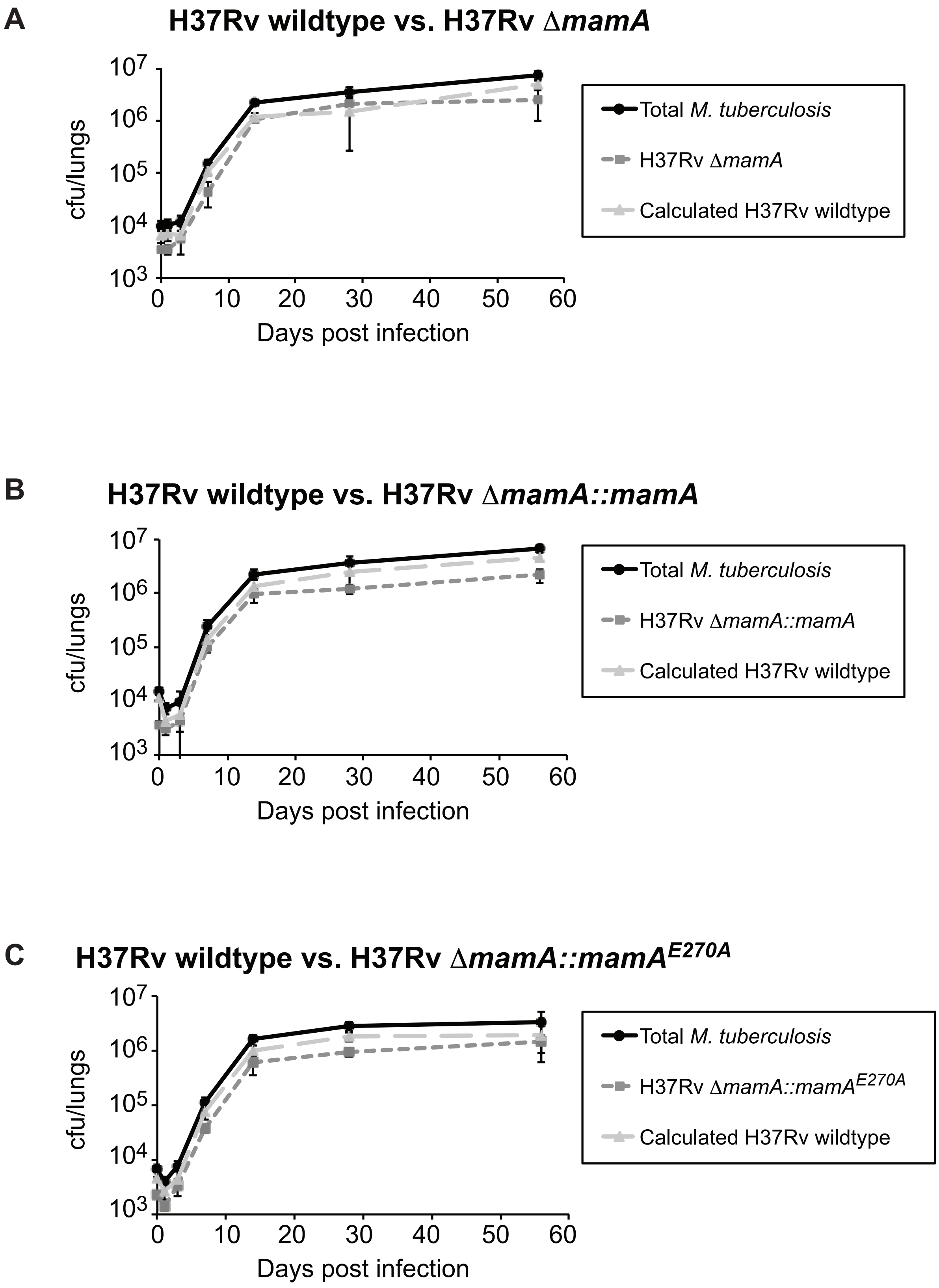
A
H37Rv wildtype vs. H37Rv ΔmamA
cfu/lungs
10^7
10^6
10^5
10^4
10^3
0 10 20 30 40 50 60
Days post infection
Total M. tuberculosis
H37Rv ΔmamA
Calculated H37Rv wildtype
B
H37Rv wildtype vs. H37Rv ΔmamA::mamA
cfu/lungs
10^7
10^6
10^5
10^4
10^3
0 10 20 30 40 50 60
Days post infection
Total M. tuberculosis
H37Rv ΔmamA::mamA
Calculated H37Rv wildtype
C
H37Rv wildtype vs. H37Rv ΔmamA::mamA^{E270A}
cfu/lungs
10^7
10^6
10^5
10^4
10^3
0 10 20 30 40 50 60
Days post infection
Total M. tuberculosis
H37Rv ΔmamA::mamA^{E270A}
Calculated H37Rv wildtype
