# Supplementary material for: DNA Methylation Impacts Gene Expression and Ensures Hypoxic Survival of Mycobacterium tuberculosis
Source: PLoS Pathog. 2013 Jul 4;9(7):e1003419. doi: 10.1371/journal.ppat.1003419 (PMC3701705; doi:10.1371/journal.ppat.1003419)
Supplement: Methods S1 — Materials and methods for experiments shown in figures S1, S2, S3, S4, S5. (DOCX) [file ppat.1003419.s007.docx]

**Methods S1**

**Determination of mutation rates.** Mutation rates were determined by fluctuation analysis. Twenty independent cultures of 2 x 10^4^ cells in 4 ml of 7H9 sup­plemented with OADC, 0.05% Tween-80 and 0.5% glycerol were grown to an OD of 1 (approximately 2 weeks) and plated on 7H10 plates with and without 2μg/ml of rifampin. The numbers of mutants on the plates were counted after 21 days of incubation at 37°C. The mutation rate was determined using the Lea and Coulson Method of the Median [1]. Rates from different strains were compared by Mann-Whitney test.

***In vitro* sensitivity to stress conditions.** To test sensitivity to reactive nitrogen species, triplicate log phase cultures were diluted to OD 0.1 and incubated for 24 hours without shaking in the presence or absence of 10 mM DETA-NO. Cultures were plated and percent survival was calculated as ((CFU/mL in DETA-NO-exposed cultures)/(CFU/ml in unexposed cultures))*100. To test sensitivity to reactive oxygen species, log phase cultures were pelleted and resuspended in 7H9 prepared without catalase or oleic acid to OD 0.2 and monitored for four days by OD measurement.

**Reference:**

1. Lea D, Coulson CA (1949) The distribution of the numbers of mutants in bacterial populations. Journal of Genetics 49: 264–285.
