# Supplementary material for: DNA Methylation Impacts Gene Expression and Ensures Hypoxic Survival of Mycobacterium tuberculosis
Source: PLoS Pathog. 2013 Jul 4;9(7):e1003419. doi: 10.1371/journal.ppat.1003419 (PMC3701705; doi:10.1371/journal.ppat.1003419)
Supplement: Table S1 — Putative MamA recognition sequences tested by sequence trace comparison. (DOCX) [file ppat.1003419.s008.docx]

**Table S1. Insert sequences tested by sequence trace comparison.**

| **Plasmid** | **Oligo or insert sequence^1,2^** | **Oligo name** | **Methylation status in H37Rv** |
| --- | --- | --- | --- |
| pSS010 | Top: GATCCGGTTTCAG**CTGGAG**CTTAGCA | SSS068 | + |
|  | Bottom: AGCTTGCTAAG**CTCCAG**CTGAAACCG | SSS069 | + |
| pSS012 | Top: GATCCTTGGGCAG**CTGGAG**CGGTAAA | SSS070 | + |
|  | Bottom: AGCTTTTACCG**CTCCAG**CTGCCCAAG | SSS071 | + |
| pSS014 | Top: GATCCGGTTTTGG**CTGGAG**CTTAGCA | SSS072 | + |
|  | Bottom: AGCTTGCTAAG**CTCCAG**CCAAAACCG | SSS073 | + |
| pSS016 | Top: GATCCGGTTTTAG**CTGGAG**CTTAGCA | SSS074 | + |
|  | Bottom: AGCTTGCTAAG**CTCCAG**CTAAAACCG | SSS075 | + |
| pSS018 | Top: GATCCTTGGGACG*CTAAAG*CGGTAAA | SSS096 | - |
|  | Bottom: AGCTTTTACCG*CTTTAG*CGTCCCAAG | SSS097 | - |
| pSS019 | Top:  GATCCTTGGGACG**CTGGAG**CGGTaacgacagagtgcAAA | SSS098 | + |
|  | Bottom:  AGCTTTTgcactctgtcgttACCG**CTCCAG**CGTCCCAAG | SSS099 | + |
| pSS020 | Top: GATCCTTGGGACG*CTGCAG*CGGTAAA | SSS100 | - |
|  | Bottom: AGCTTTTACCG*CTGCAG*CGTCCCAAG | SSS101 | - |
| pSS021 | Top: GATCCTTGGGACG*CTCGAG*CGGTAAA | SSS102 | - |
|  | Bottom: AGCTTTTACCG*CTCGAG*CGTCCCAAG | SSS103 | - |
| pSS022 | Top: GATCCTTGGGACG*CTGAAG*CGGTAAA | SSS104 | - |
|  | Bottom: AGCTTTTACCG*CTTCAG*CGTCCCAAG | SSS105 | - |
| pSS023 | Top: GATCCTTGGGACG*CTAGAG*CGGTAAA | SSS106 | - |
|  | Bottom: AGCTTTTACCG*CTCTAG*CGTCCCAAG | SSS107 | - |
| pSS024 | Top: GATCCTTGGGACG*CTATAG*CGGTAAA | SSS108 | - |
|  | Bottom: AGCTTTTACCG*CTATAG*CGTCCCAAG | SSS109 | - |
| pSS025 | Top: GATCCTTGGGACG*CTCAAG*CGGTAAA | SSS110 | - |
|  | Bottom: AGCTTTTACCG*CTTGAG*CGTCCCAAG | SSS111 | - |
| pSS026 | Top: GATCCTTGGGACG*CTACAG*CGGTAAA | SSS112 | - |
|  | Bottom: AGCTTTTACCG*CTGTAG*CGTCCCAAG | SSS113 | - |
| pSS027 | Top: GATCCTTGGGACG*CTTAAG*CGGTAAA | SSS114 | - |
|  | Bottom: AGCTTTTACCG*CTTAAG*CGTCCCAAG | SSS115 | - |
| pMV762 | CCAGTC**CTCCAG**GCG*GTCCAG*CAGCCG^3^ | None | +,-^4^ |
| pMV762 | GAGCTC**CTCCAG**GGCTCG^3^ | None | + |

^1^Oligos were annealed and cloned into BamHI, HinDIII-cut pMV762.

^2^Intact MamA sites are denoted by bold, and incomplete/disrupted MamA sites are denoted by italics.

^3^Sequences that are present in the hygromycin resistance gene in pMV762 and were not added by oligo inserts.

^4^Adenines in the bolded motif are methylated, while adenines in the italicized motif are not.
