# Supplementary material for: DNA Methylation Impacts Gene Expression and Ensures Hypoxic Survival of Mycobacterium tuberculosis
Source: PLoS Pathog. 2013 Jul 4;9(7):e1003419. doi: 10.1371/journal.ppat.1003419 (PMC3701705; doi:10.1371/journal.ppat.1003419)
Supplement: Table S3 — Mutation rates in wildtype and ΔmamA strains. (DOCX) [file ppat.1003419.s010.docx]

**Table S3. Mutation rates in wildtype and *ΔmamA* strains.**

| **Strain** | **Rif^R^ rate^a^** |
| --- | --- |
| H37Rv | 1.3 [0.52 - 2.0] x 10^-8^ |
| H37Rv ∆*mamA* | 2.2 [1.6 - 2.9] x 10^-8^ |

^a^Median rate of acquisition of resistance to rifampin with 95% confidence interval in square brackets. Rates are not significantly different by Mann-Whitney test (p>0.05).
