# Supplementary material for: DNA Methylation Impacts Gene Expression and Ensures Hypoxic Survival of Mycobacterium tuberculosis
Source: PLoS Pathog. 2013 Jul 4;9(7):e1003419. doi: 10.1371/journal.ppat.1003419 (PMC3701705; doi:10.1371/journal.ppat.1003419)
Supplement: Table S4 — Primers used for quantitative PCR. (DOCX) [file ppat.1003419.s011.docx]

**Table S4: Primers used for quantitative PCR**

| **Gene** | **Primer sequence** | **Primer name** |
| --- | --- | --- |
| *Rv0102* | Forward: CGCTACGTTCTATCGTTCC | SSS324 |
|  | Reverse: CCACGATGACCATGATGAC | SSS325 |
| *Rv0142* | Forward: GACATCGTTGCCTGGAGT | SSS316 |
|  | Reverse: CGATCATCTTCGGAATGTGG | SSS317 |
| *corA* | Forward: CTCGAGGTGACCAATCTCAT | SSS330 |
|  | Reverse: GCTTGAGCAGATAGATCGG | SSS331 |
| *sigA* | Forward: GCTGATGACCGAGCTTAG | TLR85 |
|  | Reverse: GGATCAGGTCGAGAAACG | TLR86 |
| *whiB7* | Forward: GGCTCTACGTAAGCGCTA | SSS383 |
|  | Reverse: CCTGTTTCACCTGCTTCC | SSS384 |
| *Rv3083* | Forward: CGGTCTGAACATCCAGATGC | SSS193 |
|  | Reverse: ATGACGCGTTGGTGTAGCC | SSS194 |
| *Rv3263 (mamA)* | Forward: AACTCAGCGGGCTGATCTATGACGA | SSS149 |
|  | Reverse: GAGCACTTGTAGCCGAGGTG | SSS251 |
