# Supplementary material for: DNA Methylation Impacts Gene Expression and Ensures Hypoxic Survival of Mycobacterium tuberculosis
Source: PLoS Pathog. 2013 Jul 4;9(7):e1003419. doi: 10.1371/journal.ppat.1003419 (PMC3701705; doi:10.1371/journal.ppat.1003419)
Supplement: Table S5 — Primers used for transcriptional start site mapping. (DOCX) [file ppat.1003419.s012.docx]

**Table S5: Primers used for transcriptional start site mapping**

| **Gene** | **Primer sequence** | **Primer name** |
| --- | --- | --- |
| *Rv0102* | Forward: CCCGCGAAGACCGGCATG | SSS340 |
|  | Reverse: TTGCCGGCCTCAGCGTAG | SSS347 |
| *Rv0142* | Forward: GCTTGCTCGAAGCCAGCGG | SSS338 |
|  | Reverse: ACGCAACGGCGCCAAGGTG | SSS339 |
| *corA* | Forward: GACTCCAGGTGGGGTTAC | SSS342 |
|  | Reverse: GCAGTCGACCAAGGCTTG | SSS343 |
| *whiB7* | Forward: CGGACCCGAAGTCACAGG | SSS345 |
|  | Reverse: CGACACGGTTCTTGCTATGG | SSS344 |
| *whiB1* | Forward: CATGAGCGAAGACGAGCGG | SSS311 |
|  | Reverse: AGGCGTTTCTTCTTGGCTGG | SSS310 |
